# CLL to Richter syndrome: Integrating network strategies with experiments elucidating disease drivers and personalized therapies

**DOI:** 10.1126/sciadv.adu7705

**Published:** 2025-09-12

**Authors:** Julia Maier, Julian D. Schwab, Silke D. Werle, Ralf Marienfeld, Stephan Stilgenbauer, Peter Möller, Nensi Ikonomi, Hans A. Kestler

**Affiliations:** ^1^ Institute of Medical Systems Biology, Ulm University, 89081 Ulm, Germany.; ^2^ Institute of Pathology, University Hospital of Ulm, 89081 Ulm, Germany.; ^3^ Comprehensive Cancer Center Ulm, University Hospital Ulm, 89081 Ulm, Germany.; ^4^ Division of CLL, Dept. of Internal Medicine III, University Hospital of Ulm, 89081 Ulm, Germany.

## Abstract

Chronic lymphocytic leukemia (CLL) is a common neoplasm that carries the risk of transformation into Richter’s syndrome (RS), a highly aggressive B cell lymphoma with poor prognosis. Limited availability of animal models and cell lines hinders understanding of transformation mechanisms. Addressing this gap, we established the first in silico dynamic model of the disease. Our methodology integrates mathematical logic modeling with experimental data to identify disease drivers, mechanisms, and potential therapeutic targets. We validated the model by comparing the model’s readout with experimental data from different biological levels, such as single-cell RNA sequencing analyses and a CLL/RS patient formalin-fixed paraffin-embedded (FFPE) tissue cohort. Our analyses identified BMI1 proto-oncogene and TP53 loss as key RS progression regulators. In addition, we performed an in silico target screening to identify promising target combinations in a personalized fashion. Through the synergy of mathematical modeling with experimental readouts, our model provides a complementary approach to investigate the process of CLL transformation to RS.

## Teaser

The established model was designed to identify key regulators and therapeutic targets in the transformation from CLL to RS.

## INTRODUCTION

Chronic lymphocytic leukemia (CLL) is the most common leukemia in western countries. It is an indolent B cell malignancy characterized by small, mature-appearing neoplastic lymphocytes with low mitotic activity (*1*). The cumulative acquisition of different genetic lesions may lead to progressive disease and ultimately to the development of an aggressive B cell lymphoma (*2*, *3*), called Richter syndrome (RS) (*4*, *5*), affecting 2 to 10% of patients with CLL. Most cases of RS are diffuse large B cell lymphoma (DLBCL) (*2*, *3*), but classical Hodgkin lymphoma can also be observed. Despite intensive therapy (*6*, *7*), the outcome of RS is still dismal, and most patients succumb to the disease within the first year after transformation (*8*–*10*). RS must be distinguished from an “acceleration” of CLL with expansion of proliferation centers (*11*). The DLBCL variant of RS displays an immune phenotype different from the underlying CLL, showing loss of the surface markers CD5 and CD23 are lost in most cases of RS, while the expression of CD19 is invariably maintained (*12*).

Multiple clinical and biological risk factors leading to the development of RS have been unraveled, including nonmutated immunoglobulin heavy chain variable region (*IGHV*) (*9*, *13*) or mutated Notch receptor 1 (*NOTCH1*) (*14*, *15*), as well as mutation or deletion of tumor protein p53 (*TP53*) (*3*, *16*). Although RS was shown to be a heterogeneous disease with differences in the underlying genetic profile (*9*, *17*), aberrations in *TP53*, MYC proto-oncogene (*MYC*), *NOTCH1*, or cyclin dependent inhibitor 2A (*CDKN2A*) are commonly found in RS, thus affecting regulators of apoptosis and proliferation (*18*, *19*).

Recent studies using large-scale genetics, transcriptomics, and epigenomics in patient cohorts disclosed several pathways to be active in RS, affecting DNA damage, mitogen-activated protein kinase (MAPK), MYC signaling, chromatin regulation pathways, nuclear factor κB (NF-κB), and phosphoinositide 3-kinase (PI3K) signaling, as well as oxidative phosphorylation (*17*, *20*, *21*).

While a crucial role of the tumor microenvironment (TME) for CLL initiation and progression is well known (*1*), TME contribution to the transformation process to RS is yet incompletely understood. In lymph node and bone marrow, cellular interactions with the TME are pivotal for driving the proliferation of the tumor cells (*22*–*24*), while in the peripheral blood, CLL cells show a mainly anergic nonproliferating phenotype (*25*, *26*). Noteworthy, CLL and RS differ in the underlying TME processes as the immune cell composition changes during tumor evolution (*27*).

The mechanistic understanding of Richter’s transformation is highly limited by the availability of suitable preclinical models for RS (*28*). Substantial advances have been made in the generation of genetically engineered mouse models (*29*–*32*), patient-derived xenograft (PDX) models (*33*, *34*), and in vitro cell lines (*35*). However, these strategies still struggle to capture the TME or reflect inter- and intrapatient tumor heterogeneity.

To address the complexity and diversity of the disease and its potential multistep pathogenic processes, we have adopted an integrated systems biology approach to better understand the mechanism of transformation and to identify disease drivers and drug targets. To this aim, we constructed a mathematical model relying on Boolean logics involving the main molecular pathways and its cross-talks in CLL. Boolean models have already been widely used to provide robust predictions on disease drivers and drug targets in a variety of biomedical applications (*36*). In this work, we furthermore integrate mathematical modeling with experimental results from a CLL/RS patient cohort and published single-cell RNA sequencing (scRNA-seq) data to develop a strategy to study the aggressive transformation of CLL to RS.

## RESULTS

### Establishing a mathematical model of CLL: Construction and stability assessment

**Fig. 1.**
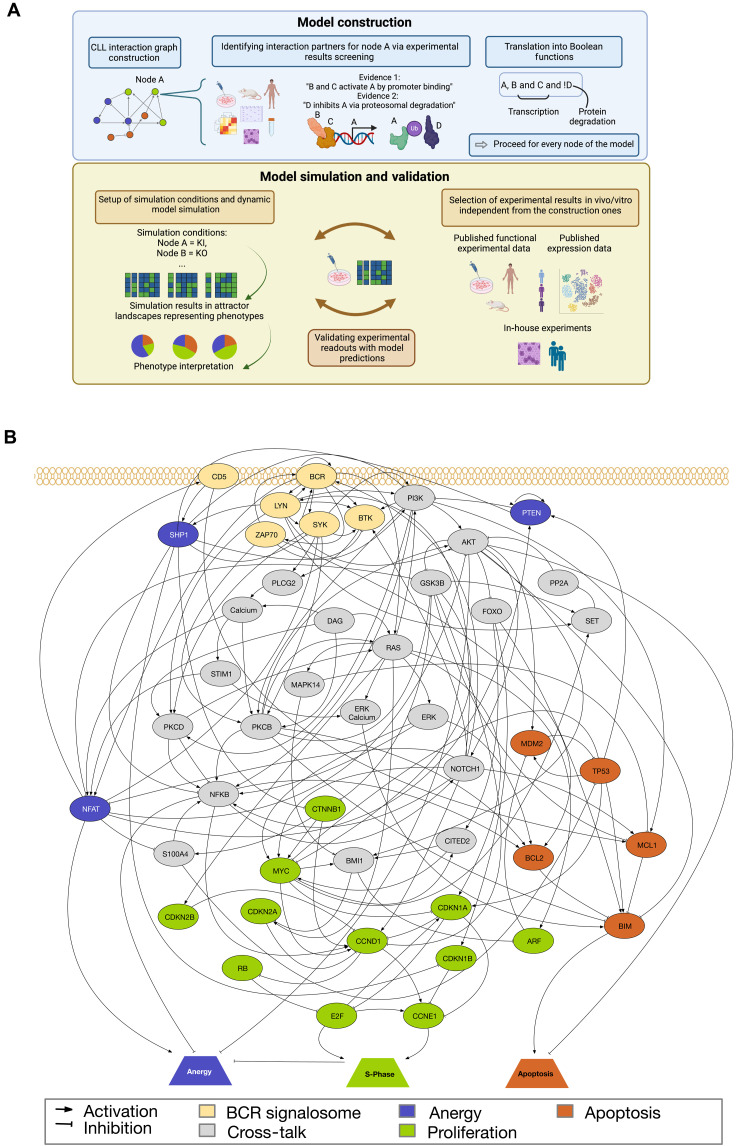
Visualization of the CLL model construction, validation, and interactome. (**A**) Figure depicts the workflow of model construction and validation. After gathering experimental information from literature and databases, a preliminary interactome of the model is integrated (light blue part). For each node in the model, literature statements are integrated to generate Boolean regulatory functions using logic gates (AND, OR, and NOT). Once Boolean regulatory functions are implemented, the model is validated on the basis of dynamic simulations (light yellow part). Here, independent experiments from literature, omics datasets, or in-house data are used. Validation considers readout nodes and internal nodes of the model. (**B**) Interaction graph underlining the final CLL logic model. Nodes are shown as circles, and indicator nodes are shown in trapezes. Interactions are depicted as pointed arrows for activatory regulations and bar-headed ones for inhibitory ones. Nodes are colored according to the biological process modeled. Respectively, BCR signalosome (light yellow), anergy (dark purple), apoptosis (orange), proliferation (light green), and cross-talk nodes in gray. K, knockin; KO, knockout.

**Fig. 2.**
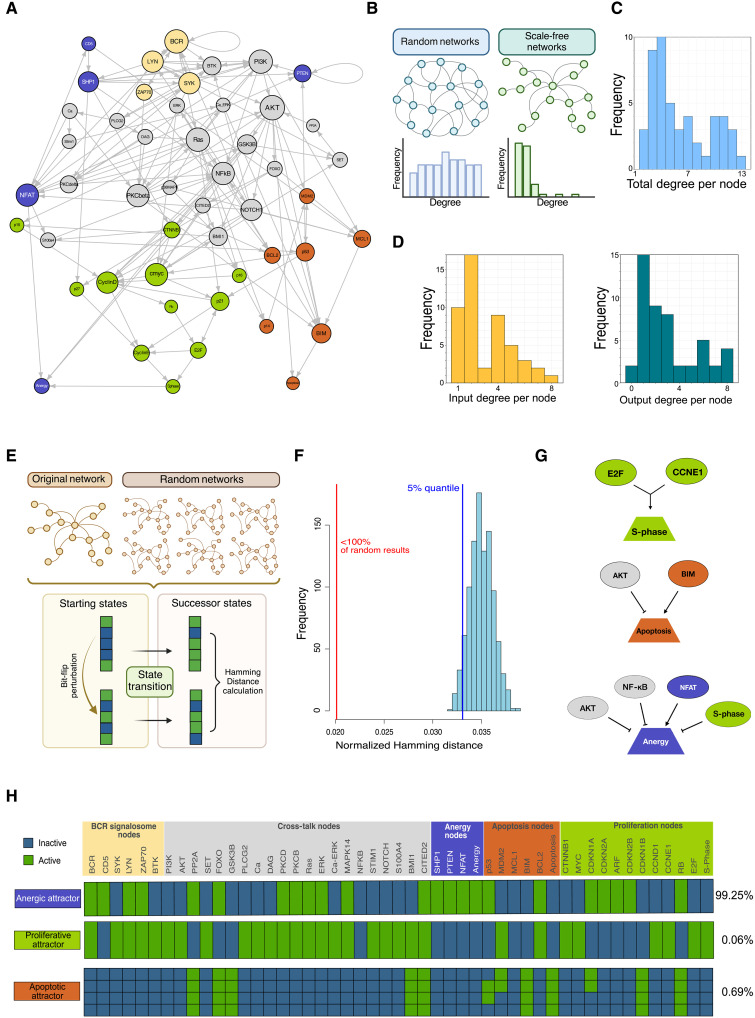
Network theoretical interaction-graph based and dynamic properties. (**A**) Interactome of the CLL model. Nodes sizes indicate the degree of connectivity within the network. (**B**) General visualization of scale-free networks. Biologically motivated models show scale-free topologies (in green), where nodes show a high variance in the degree of connectivity. Random networks show an equally distributed amount of connectivity among all nodes. (**C**) Total degree per node in the CLL model is depicted in form of a histogram showing the degree level and the frequency that degree within the model. (**D**) In a similar manner, the input and output degree per node is depicted. (**E**) General outlook of the stability test to random perturbations which assesses the stability of the CLL models toward bit-flip perturbations. Specifically, a starting state of the network receives one bit-flip perturbation. Then, the next state that is simulated for both original and perturbed initial states is computed, and the normalized Hamming distance between the two is calculated. The procedure is performed for both the CLL model and a set or randomly generated networks with similar structural properties. (**F**) One thousand randomly drawn states of the CLL model were used for the analysis. The normalized hamming distance of the CLL model states is indicated in red. The same analysis was performed for 1000 randomly drawn models (light blue histogram), and the blue line indicates the 5% quantile. (**G**) Indicator nodes and their direct regulators are depicted. (**H**) Attractor landscape of the unperturbed CLL model. Phenotypical interpretation of the attractors is provided on the left within the squared boxes. The basin size estimation of each attractor is reported on the right as percentage. Blue boxes within the attractor patterns indicate inactive nodes (OFF), and green boxes indicate active ones (ON).

To unravel the dynamic mechanisms behind CLL and RS, we established a mathematical model based on logic regulatory functions (Fig. 1). Nodes and their interactions represent genes and cross-talks frequently altered in CLL, resulting in a final dynamic model comprising 49 nodes and 147 interactions. The biological information underlying the model construction was based on different experimental results from 228 independent publications. All underlying papers are peer-reviewed, and literature research was conducted using National Center for Biotechnology Information (NCBI), Google Scholar, and Metacore (Clarivate). The experimental results found in these papers provided the grounds for constructing one Boolean rule for each of the 49 genes of our CLL network. When available, studies from different CLL mouse models, human peripheral blood CLL cells, CLL cell lines, or non-neoplastic B cells were prioritized. The methods part of this work provides a detailed strategy on how the Boolean rules were constructed. A visual summary of the modeling strategy is shown in Fig. 1A. Also, a detailed description of the Boolean functions is available in table S1. Before exploring the system’s dynamics, we assessed whether the constructed model reflects well-known features of biologically motivated networks (Fig. 2, A to F), such as scale-free topology and resistance to random noise. Both properties aim to assess the robustness of the modeled cross-talk. We tested both properties and could confirm that our model does show scale-free properties (*P* = 0.47, significance a7 *P* > 0.1). Thus, it fits a power law distribution representing a few highly connected hub nodes within the network structure (Fig. 2, B to D). Our model also exhibits resistance to random bit flip perturbations compared to randomly generated models (Fig. 2, E and F). Here, the measured normalized Hamming distance for the CLL model was indeed smaller than the 5% quantile of the randomly generated ones, with the origin of the CLL model at 0.02 while 5% quantile of the randomly generated ones at 0.03 (Fig. 2F).

### Model simulations predict known traits of CLL cellular behavior

**Fig. 3.**
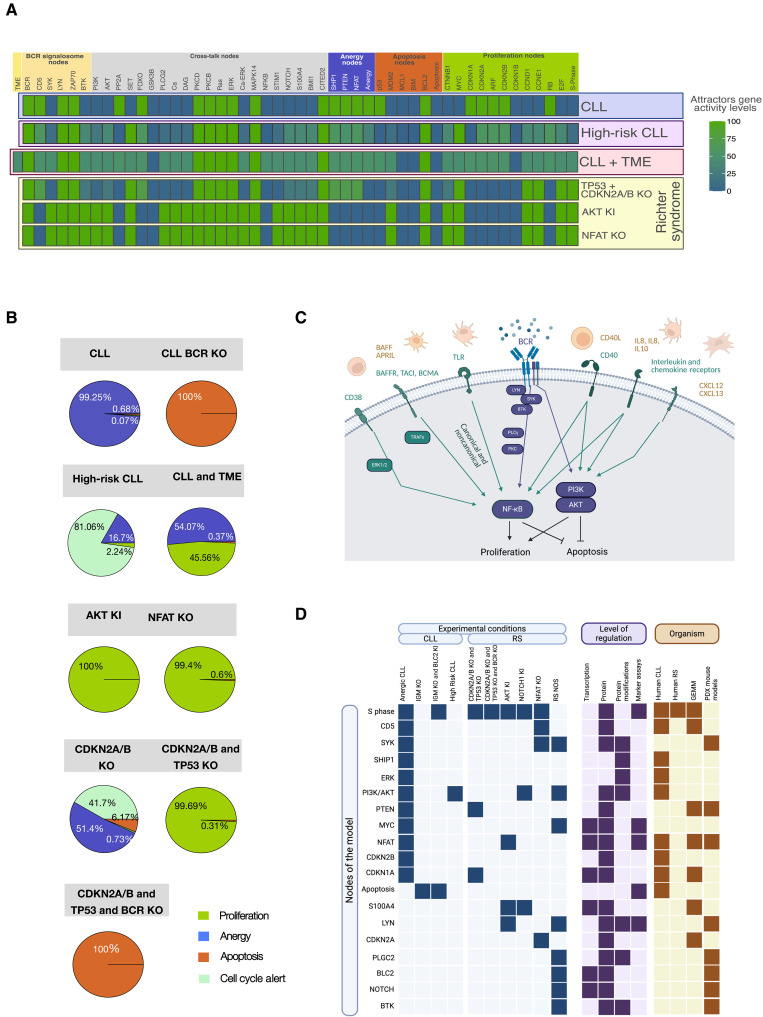
Model predictions and their validation with published experimental results. (**A**) Figure shows the attractor landscapes of the CLL and RS conditions. The specific condition is indicated on the right grouped by colors depending on CLL, high-risk CLL, TME extension, or RS conditions. For each node, the activity is indicated as the averaged percentage of node activity within the attractors weighted by the basin estimations. Blue indicates inactive nodes, while green indicates active ones. Nodes are grouped according to their pathway association. (**B**) Attractor patterns of the simulated CLL and RS conditions are depicted in the form of pie charts. Each attractor is matched to a phenotypical readout and assigned to a color according to the provided legend. Percentages of each phenotype indicated the size of the basin of attraction for each retrieved phenotypical readout within the attractor patterns. (**C**) Graphical representation of the CLL microenvironment. External stimuli are represented outside the cell. The corresponding receptors are indicated on the cellular membrane. Last, the stimulated nodes are depicted as circles. Phenotypical readouts connected to the network nodes are represented on the bottom. (**D**) Graphical representation of the model validation by published experimental readouts. Nodes evaluated within the validation are depicted on the left as rows. Columns are grouped according to experimental condition (CLL or RS), level of regulation in which the experiment was performed (transcription, protein, protein modifications, marker assays), and organism (human CLL and RS or mouse models). Filled darker squares indicate a hit for a node within the specific condition, level of regulation, and model system.

After constructing the model, we further moved to validating its dynamic behavior. To do so, we simulated different CLL conditions and compared our simulations with experimental results by phenotypical traits in terms of proliferation, cell survival, and gene-specific activities (Table 1 and Figs. 2H and 3D).

**Table 1.** Model validation with published experimental results. The table shows the validation procedure of the CLL model. The model conditions are divided based on CLL- or RS-related phenotypes. For each phenotype, the specific modeling condition is reported. For the RS not otherwise specified (NOS) condition, the experimental readout was compared to all three RS simulation subtypes. Nodes were considered as matching if at least two of the three RS subtypes show the same readout as the experimental data. The readout activity within the model used for validation purposes is also shown. The associated experimental evidence is presented with the exact experiment confirming the model activity, together with the model organism/human cohort in which it was performed. References to published results are provided as Pubmed IDs. RT-qPCR, reverse transcription quantitative polymerase chain reaction; BrdU, 5-bromo-2′-deoxyuridine; H&E, hematoxylin and eosin; PDX, patient-derived xenograft.

Model condition		Node (model-based status)	Experimental evidence for model-based status	Model system	Reference (Pubmed ID)
**CLL phenotype**	Anergic CLL	S phase (OFF)	Ki67 immunohistochemistry	Del 13q GEMM CLL mouse model	36468984
		CD5 (ON)	Immunocytochemistry for CD5	Human CLL cells	8104532
		SYK (OFF)	Immunoblot of p-SYK/SYK	Eu-TCL1 mouse model	28970470
		SHP1 (ON)	Immunoblot for p-SHP1/SHP	CLL PBMC human	28619847
		ERK (ON)	Immunoblot for p-ERK/ERK	CLL PBMC human	28970470, 18292287
		PI3K/AKT (OFF)	Immunoblot for p-AKT/AKT	CLL PBMC human	18292287
			Phospho-immunofluorescence	CLL PBMC human	33538798
			Immunoblot for p-AKT/AKT	Eμ-TCL1 mouse model	33538798
		PTEN (ON)	Immunoblot for PTEN	Del 13q GEMM CLL mouse model	36468984
		c-myc (OFF)	Immunohistochemistry and immunoblot for c-myc	Del 13q GEMM CLL mouse model	36468984
		NFAT (ON)	Immunoblot, qPCR and nuclear translocation of NFAT	CLL lymph node human	28970470
			Nuclear localization and DNA binding of NFAT	CLL PBMC human and CLL cell lines	18292287
		CDKN2B (ON)	Immunoblot for CDKN2B after IgM stimulation	CLL PBMC human	33900379
		CDKN1A (ON)	Immunoblot for CDKN1A	Eμ-TCL1 mouse model	33900379
			RT-qPCR for CDKN1A	Human CLL cells	33900379
	IgM KO	Apoptosis (ON)	Cell viability assay upon IgM deprivation ex vivo	CLL PBMC human	8104532
			Percent apoptotic cells after IgM stimulation ex vivo	CLL PBMC human	26924423
	IgM KO and BCL2 KI	S phase (OFF), Apoptosis (OFF)	Cell viability dependance on BCL2 levels upon IgM deprivation	CLL PBMC human	8104532
	High risk CLL	AKT (partially ON)	Immunoblot for p-AKT/AKT	CLL PBMC human	33538798, 30559170
	CDKN2A/B KO	S phase (partially ON)	Count of leukemic cells	Eμ-TCL1^CDKN2A/B−/−^ mouse model	33900379
**RS phenotype**	CDKN2A/B KO and TP53 KO	S phase (ON)	Leukemic cells overgrowth, blastic transformation in H&E histochemistry, BrdU incorporation	Eμ-TCL1^CDKN2A/B/TP53−/−^ mouse model	33900379
		c-myc (ON)	No growth advantage after additional MGA KO (c-myc inhibitor)	Eμ-TCL1^CDKN2A/B/TP53−/−^ mouse model	33900379
		CDKN1A (OFF)	Immunoblot for CDKN1A	Eμ-TCL1^CDKN2A/B/TP53−/−^ mouse model	33900379
	CDKN2A/B and TP53 and BCR KO	S phase (OFF)	Count of leukemic cells	Eμ-TCL1^CDKN2A/B/TP53/IgM−/−^ mouse model	33900379
			BrdU incorporation upon IgM deprivation	Human RS cells with CDKN2A/B and TP53 disruption	33900379
	AKT KI	S100A4 (ON)	RT-qPCR for S100A4 and Proteomic analysis	Eμ-TCL1^AKT-C^ mouse model	33538798
		S phase (ON)	Massive Splenomegaly and blastic transformation in H&E histochemistry, Ki67 immunohistochemistry. Proteomic analysis via CDK1 protein expression.	Eμ-TCL1^AKT-C^ mouse model	33538798
		Lyn (ON)	Phosphoproteomic analysis and kinase scores	Eμ-TCL1^AKT-C^ mouse model	33538798
		NFAT (OFF)	Proteomic analysis	Eμ-TCL1^AKT-C^ mouse model	33538798
	NOTCH1 KI	Proliferation (S phase)	Massive Splenomegaly and blastic transformation in H&E histochemistry	Eμ-TCL1^NOTCH1-1C^ mouse model	33538798
		AKT (ON)	Immunoblot of p-AKT /panAKT	CLL PBMC human	33538798
		S100A4 (ON)	RT-qPCR for S100A4	Eμ-TCL1^NOTCH1-1C^ mouse model	33538798
	NFAT KO	S phase (ON)	Cell cycle analysis via flow cytometry	Eμ-TCL1^NFAT−/−^ mouse model	28970470
		SYK (ON)	Immunoblot of p-SYK/SYK	Eμ-TCL1^NFAT−/−^ mouse model	28970470
		CDKN2A (OFF)	Immunoblot for CDKN1A	Eμ-TCL1^NFAT−/−^ mouse model	28970470
		CD5 (OFF)	Flow cytometry for CD5	Eμ-TCL1^NFAT−/−^ mouse model	28970470
	RS NOS	PLCG2 (ON)	Immunoblot for p-PLCG2 and PLCG2	RS PDX mouse model	33654205
		MYC (ON)	Immunoblot for c-MYC, RT-qPCR and RNA-seq	Different RS PDX mouse model	33654205, 38017105, 29735551
		AKT (ON)	Immunoblot for p-AKT/AKT	RS PDX mouse model	33654205
		BTK (ON)	Immunoblot for p-BTK/BTK	RS PDX mouse model	33654205, 29735551
		BCL2 (ON)	Immunoblot for BCL2 and RNA-seq	Different RS PDX mouse models	33654205, 38017105
		NOTCH (ON)	Immunoblot and RT-qPCR for NOTCH1	RS PDX mouse model	29735551
		LYN (ON)	Immunoblot for p-LYN/LYN	RS PDX mouse model	29735551
		SYK (ON)	Immunoblot for p-SYK/SYK	RS PDX mouse model	29735551

Our unperturbed CLL simulation considers the tumor in the context of B cell receptor (BCR) stimulation without costimulatory signals from the TME. The main attractor of this simulation shows activation of multiple downstream pathways involving protein kinase C (PKC) and MAPK as well as inhibitory proteins such as protein tyrosin phosphatase-1 (SHP1) and phosphatase and tensin homolog (PTEN). This activity pattern leads to an anergic cell state with active nuclear factor–activated T cells (NFAT) and extracellular signal–regulated kinase (ERK) but inactive NF-κB and protein kinase B (AKT) (Figs. 2H and 3A). Our attractor patterns show low cellular responsiveness to immunoglobulin M (IgM) stimulation in terms of proliferation, consistent with only small fractions of the proliferating phenotypes (Fig. 3, A and B).

As another CLL-related condition, we considered a high-risk CLL simulation setup with in silico knockin (KI) of MYC and tyrosine-protein kinase ZAP-70 (ZAP70) and knockout (KO) of TP53 (Fig. 3A and fig. S2G). This simulation resulted in attractor patterns in which ~16% of the estimated basin of attraction remained anergic, a minority of ~2% showed single-state attractor proliferation, and the rest of the phenotypical landscape was characterized by unstable attractors cycling between a cell cycle alert and proliferation. In both conditions, CLL attractor landscapes become apoptotic after withdrawal of IgM stimulation (Fig. 3B).

Because microenvironmental influences are known to be a crucial driver of proliferation in CLL, we implemented signals from the TME as an extension to our initial model. This was done by creating an input node “TME,” which activates BCR and AKT and NF-κB signaling (Fig. 3C). The simulation of the unperturbed CLL model, including the TME, then led to a more heterogeneous attractor landscape, showing 47% of proliferation and 53% of states staying in an anergic phenotype (Fig. 3, A and B, and fig. S2H).

### Targeted perturbation of nodes can successfully induce RS-like attractors

For the RS conditions, we matched our RS dynamic simulation predictions to three recently published mouse models by Chakraborty *et al.* (*37*), Kohlhaas *et al.* (*38*), and Märklin et al. (*26*), where RS-like phenotypes were respectively observed after *CDKN2A/B* and *TP53 KO*, *NFAT KO*, or constitutive AKT activation.

Chakraborty *et al.* (*37*) describe the development of an RS-like phenotype in a CLL mouse model with high proliferative activity independently from costimulation. The authors observed a reduction of cyclin-dependent inhibitor 1A (CDKN1A) protein levels after KO of *CDKN2A/B* and *TP53*. Furthermore, they show that the aggressive tumor is still reliant on IgM and that KO of *CDKN2A/B* alone with *TP53* wild type (WT) does not lead to RS in vivo. In addition, constitutive MYC activation shows no survival advantage in their mouse model.

Our model simulation results of CDKN2A/B and TP53 KO are depicted in Fig. 3 (A and B) and fig. S2 (A to C). We observed a proliferating attractor in 99% of the simulated starting conditions. Consistent with experimental results, proliferation depends on active BCR, confirmed by the induction of apoptosis upon BCR in silico KO. The reduction of CDKN1A protein levels after KO of CDKN2A/B and TP53 is also displayed in our model. Furthermore, CDKN2A/B KO with unperturbed TP53 leads to a small fraction of the synthesis phase (S-phase) attractor, ~50% of an anergic attractor, and the rest of the states in a cell cycle alert/Gap 1 (G1) restriction phase, again showing that our model matches the experimentally observed phenotypes. Because MYC is active already across all proliferative attractors of the CKDN2A/B and TP53 KO model, an additional constant activation of MYC did not show any difference consistent with what Chakraborty *et al.* (*37*) observed.

As a second RS condition, we performed in silico AKT KI. As recently published by Kohlhaas *et al.* (*38*), constitutively active AKT leads to RS phenotype in the CLL mouse model. They describe that these RS-like cells lose their NFAT activity and show high expression levels of S100 calcium-binding protein A4 (S100A4). In our CLL model, an in silico AKT KI confirmed these and other observations the authors describe (Fig. 3, A and B; fig. S2D; and Table 1).

For the third RS condition we investigated, we simulated in silico KO of NFAT, which is known as a critical regulator of anergy in CLL. As shown by Märklin *et al.* (*26*), NFAT loss leads to an RS phenotype in the CLL mouse model with loss of the cell cycle inhibitors CDKN2A-p14 and TP53 and the activation of AKT. BCR stimulation induced tyrosine-protein kinase SYK (SYK) phosphorylation in the NFAT-deleted cohort, while anergic, NFAT-expressing CLL cells showed any evidence of SYK phosphorylation after IgM stimulation. Again, all these findings can be observed in the NFAT in silico KO of our CLL model (Fig. 3, A and B, and fig. S2F). The loss of NFAT leads to a nearly complete proliferative attractor, which shows the activation of AKT signaling and the loss of CDKN2A.

### Gene-wise validation with experimental data confirms predictions for RS/CLL phenotypes

To thoroughly validate the CLL model and the different RS conditions with experimental readouts, we used published experimental data assessed from primary CLL cells, CLL cell lines, different PDX models [Vaisitti *et al.* (*33*), Fiskus *et al.* (*39*), and Playa-Albinyana *et al.* (*34*)], genetically engineered mouse models (GEMMs) based on the TCL1 mouse model [Kohlhaas *et al.* (*38*), Chakraborty *et al.* (*37*), and Märklin *et al.* (*26*)] and GEMM based on 13q CLL mouse model [Ten Hacken *et al.* (*32*)]. We also tested different levels of regulation, specifically mRNA expression levels [RNA-seq and reverse transcription quantitative polymerase chain reaction (RT-qPCR)], protein expression levels (immunoblot, immunohistochemistry, and proteomics data), protein modifications (phospho-immunoblot and phospho-proteomics), and various functional readouts such as nuclear translocation of transcription factors, DNA binding of transcription factors, and assessment of cell viability and apoptosis through cell cycle analysis, 5-bromo-2′-deoxyuridine (BrdU) incorporation, or Ki67 immunohistochemistry.

Most of the model validation was done on a gene-wise/node-wise level. For example, we observed an activation of NFAT in our anergic CLL model simulations. Also, Märklin *et al.* (*26*) showed high expression levels of NFAT assessed by immunoblot and RT-qPCR in human anergic CLL from peripheral blood mononuclear cells (PBMC) (*26*). In addition, Muzio *et al.* (*40*) described nuclear translocation of NFATc1 and consecutive DNA binding of the transcription factor (*40*). In this manner, we were able to validate the activation of the active status of the node NFAT in our anergic CLL model. In addition to this gene-wise approach, we also validated on the level of our integrated indicator nodes S-phase, apoptosis, and anergy. To mention an example, Chakraborty *et al.* (*37*) and Kohlhaas *et al.* (*38*) showed blastic transformation in hematoxylin and eosin (H&E) histochemistry, BrdU incorporation, high Ki67 staining, and CDK1 protein overexpression in proteomic analyses in their genetically engineered mouse models with RS-like phenotype. These experiments we used to confirm active S-phase in our equivalent in silico perturbation models, which also showed RS-like phenotypes with active S phase.

In addition, we compared our transcriptionally regulated module of nodes in the CLL-attractors to the large patient cohort of ~600 samples provided within the CLL-map portal (*41*). Here, 9 of the 13 nodes show a tendency to match the expression pattern of the CLL-map cohort (see fig. S7).

The experimental data used to validate the model were independent and different from the data we used to construct it. A detailed insight into the validation results is provided in Table 1. Figure 3D gives a visual overview about different levels of regulation and model systems used.

### Prediction of CD5 loss is validated in a patient cohort

**Fig. 4.**
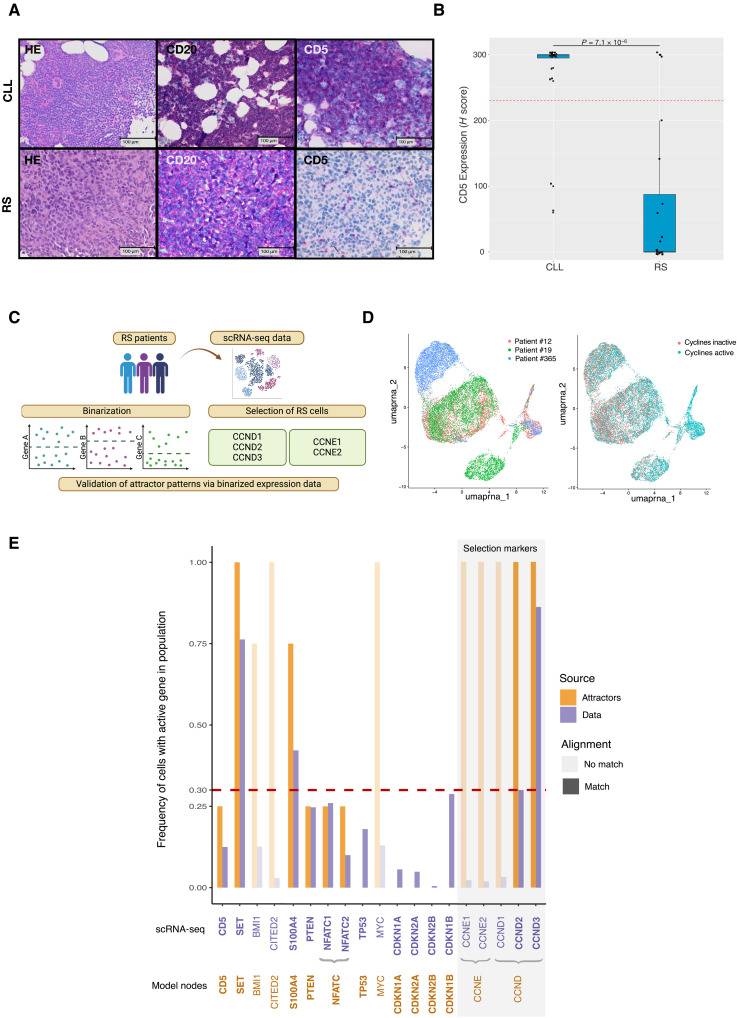
Model validation with in-house patient cohort and sc-RNA seq data. (**A**) Example of H&E and immunohistochemistry stainings for CD20 and CD5 in one CLL and one RS patient within the cohort. (**B**) *H*-score levels of CD5 stainings within the patient cohort. Patients are grouped in CLL (*n* = 28) and RS (*n* = 23) cases. Boxplots depict the median and first and third quartiles. A Wilcoxon test was applied between the two groups (*P* < 0.05 for significance). The dashed red line indicates the binarization threshold, defining the value separating the conditions simultaneously with the highest sensitivity and specificity. (**C**) Graphical visualization of the scRNA-seq data validation. The scRNA-seq data were binarized by gene of interest and then selected for RS cells depending on the activity of either one of the cyclin D and E isoforms. Last, the binarized expression data were compared to the attractor patterns. (**D**) UMAP plots of the three different patients and the binarized activities of the cyclins. (**E**) Validation of the attractor patterns with the scRNA-seq data. The frequency of an active node within our simulated RS conditions is depicted in orange. In purple, the corresponding frequency of the cells within the population of the single cell data shows active gene expression. The intensity of the colors within the histograms indicates whether the activities were found in accordance or discordance between model and data. The dashed red line indicates the threshold for considering a node/gene active (according to the Hans score). A light gray box highlights the selection markers that extract the cells. Last, nodes of the model and their corresponding genes, including isoforms within the data, are depicted below and colored accordingly.

The simulation of the model revealed that the anergic CLL attractor shows active CD5. Instead, CD5 activity is completely lost in the AKT KI and the NFAT KO perturbations and partially in the CDKN2A/B and TP53 KO attractors. To validate this pattern, we conducted CD5 and CD20 immunohistochemistry staining on formalin-fixed paraffin-embedded (FFPE) slides from 20 CLL and 20 RS tissue specimens from our patient cohort (Fig. 4, A and B). The evaluation of the staining showed that CD5 expression is lost upon transformation of the CLL into RS in most tumors. To compare these results with our in silico model activity, we binarized the expression values (fig. S3) and found that the CD5 activity in our model simulations reflects the extent of CD5 expression in the cohort. The reported loss of CD5 expression upon Richter’s transformation reflects the current state of knowledge, again showing that the model can reproduce known behaviors of the tumor.

### Model validation using scRNA-seq addresses its applicability to tumor heterogeneity

After evaluating the attractor landscapes in published data and our FFPE cohort, we further challenged our modeling strategy in evaluating and interpreting scRNA-seq data. Here, we used scRNA-seq data from three patients with RS recently published in the work of Nadeu *et al.* (*21*). To compare the single-cell expression with the model readouts, we designed a specific strategy to compare the transcriptionally regulated gene activities obtained in the model attractors with the pooled expression data of the three patients (Fig. 4C). After binarizing the single-cell expression data and filtering for proliferative cells (Fig. 4, C and D), we quantified the percentage of activation within the cell population and the corresponding percentage of activation in our RS simulated conditions (Fig. 4E). As a threshold for considering the specific gene as overall active or inactive, we used a threshold of 30%, corresponding to what is defined by the Hans score (*42*) used in context of histopathological subtyping of diffuse large B cell lymphoma. Notably, of the 13 nodes included in the transcriptional regulatory core, 10 matched the attractor predictions. Only three nodes did not show congruency between patient expression data and attractors, namely, MYC, Cbp/P300 interacting transactivator 2 (CITED2), and BMI1 proto-oncogene (BMI1).

**Fig. 5.**
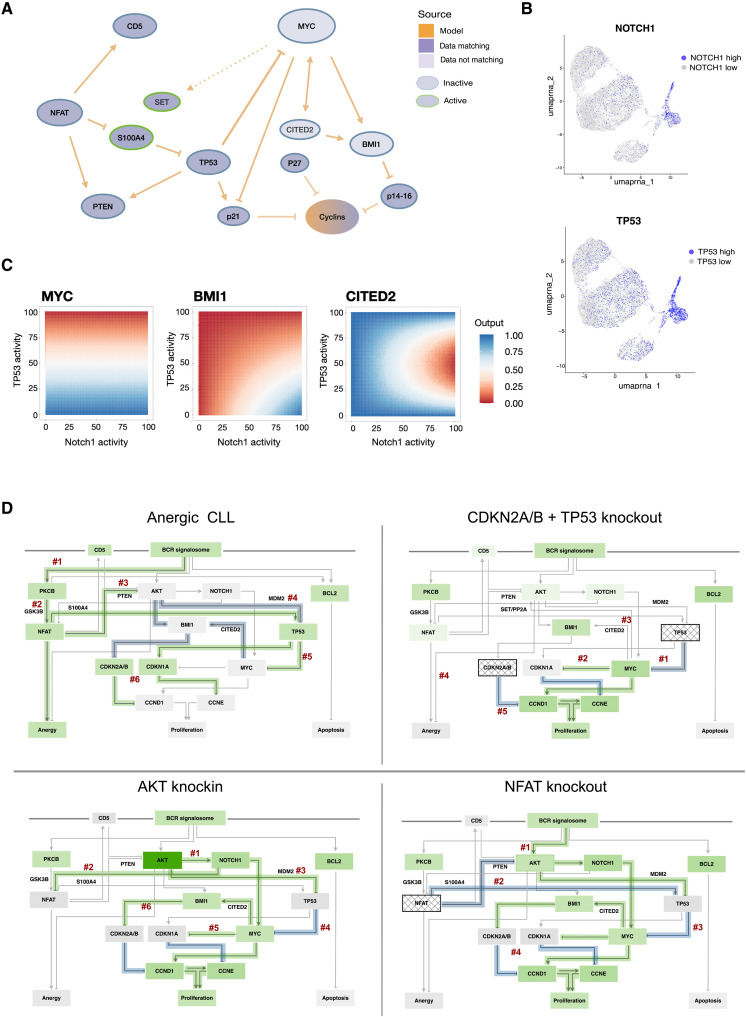
Evaluation of the transcriptional interactome and model-hypothesized mechanisms. (**A**) Interactome within the CLL model of the transcriptionally regulated genes. Nodes are indicated within circles. Dark violet circles indicate nodes matching the data, while light purple ones indicate nodes not matching. Colored borders indicate active (green) and inactive (blue) nodes. Orange indicates information coming from the CLL model. Pointed arrows indicate activation, while bar-headed ones indicate inhibition. Dashed arrows indicate minor regulations within the model. (**B**) UMAP plots of the three pooled patients, marking respectively cells with high NOTCH1 and high TP53 expression levels. (**C**) Input-output analysis results for the three nodes MYC, BMI1, and CITED2. The nodes used as inputs (NOTCH and TP53) are indicated on the axes and distributed between 0 and 100% of active states. For each point of the heatmap, the resulting level of the recorded output node is depicted as activity level between 0% and 100% of the examined trajectory of states. High levels of output activities are indicated in blue, medium levels in white, and low activity in red, as also indicated in the legend. (**D**) Regulatory circuits summarizing the trajectories leading to CLL and RS attractors of the model are depicted. Here, principal regulations and nodes are reported. Green boxes around nodes indicate stable activity, while light green boxes indicate oscillating activity. Light green arrows indicate the regulation is taking place under certain conditions. Blue arrows indicate that regulation is impeded. Arrows indicate activation interactions, and bar-headed lines indicate inhibitory interactions. KOs are gray gridded boxes, while constitutive activation (KI) is dark green.

A deeper investigation on the biologic features of the scRNA-seq patient cohort (*21*) revealed that Pt#12 harbors a *TP53* mutation, which can be classified as mutation class 3, variant of unknown significance (VUS) according to ClinVar (*43*) and Varsome (*44*) databases. However, tumor cells of Pt#19 harbor three different *TP53* mutations, which can all three be assigned to class 4 or 5 mutations (ClinVar, Varsome). Pt#365 shows WT *TP53*. Recapitulatory, neglecting the VUS mutation of Pt#12, only Pt#19 shows a potentially oncogenic TP53 lesion with a maximal 25% of cells affected by a biallelic loss of function. Together, the analyzed cohort shows a rather functional *TP53*. This is also reflected by the uniform manifold approximation and projection (UMAP) plots in Fig. 5B, where the proliferating part of the cells also shows high mRNA levels of TP53.

The high levels of *TP53* stand in contrast to our simulated RS subtypes since one subtype is already simulated with a TP53 KO, and the other subtypes show at least a partial loss of activity during the trajectory to the RS attractor.

In addition, two patients harbor an activating *NOTCH1* mutation, which is again reflected in the UMAP plots, where the *NOTCH1* active areas essentially correspond to the active cyclin areas (Fig. 5B).

Considering this information, we reconstructed the CLL subnetwork of the analyzed transcriptionally regulated nodes (Fig. 5A) and found that, according to our model, TP53 strongly inhibits transcriptional activation of *MYC*, which itself further activates *CITED2* and *BMI1* on a transcriptional level. The influence of MYC as an enhancer of transcription on SET nuclear proto-oncogene (*SET*), however, is weak (*45*) because *SET* expression is also activated by glycogen synthase kinase 3 beta (GSK3B) and LYN proto-oncogene (LYN). Also, MYC strongly represses *CDKN1A* expression, leading to low levels of CDKN1A independent of TP53 activity status.

Together, the connectivity between TP53, MYC, BMI1, and SET as derived from our model rules explains the observed up-regulation of MYC, BMI1, and SET in our attractor landscape compared to the scRNA-seq data.

To better capture the specific genetic features of the scRNA cohort, we further investigated our system from a more quantitative perspective. Thus, we performed an input-output analysis by studying “NOTCH1” and “TP53” as inputs. As outputs, we primarily focused on the nodes BMI1, CITED2, and MYC since their activity was discordant with the binarized mRNA levels in the patient data. Here, all three nodes of interest showed a dependence on TP53 signal intensities for their induction (Fig. 5C). In detail, high activity levels of TP53 lead to low expression of MYC independent from NOTCH1 activity. Also, BMI1 activity levels drop with increasing levels of TP53, thus representing the heterogeneity in behaviors observed in the binarized data. Noteworthy, BMI1 activity is also increasing with high NOTCH1 levels. CITED2 can only be active together with either very strong or very low levels of TP53. Also, a high activity of NOTCH1, as expected in the scRNA-seq patient cohort, will lead to inactive CITED2. Overall, simulations that include the extension to the input-output relations could reliably recapitulate the overall expression data and capture spots of tumor heterogeneity.

### Tumor trajectories unravel the mechanisms leading to RS attractors

To understand the underlying mechanisms of the predicted phenotypes, we examined tumor trajectories by studying the dynamic cascades from BCR-stimulated starting conditions to the stable states of the systems. Our results revealed differentially involved regulatory circuits described below (Fig. 5D and fig. S4).

It was shown that IgM stimulation of CLL results in the expression of cell cycle inhibitors (*37*). Using our tumor trajectories, we can now add mechanistic insights to this observation (Fig. 5D, top left). Upon BCR stimulation, PKC beta (PKCB) gets activated (#1). This is followed by the inhibitory phosphorylation of GSK3B, which, in turn, allows the activation of NFAT (#2). NFAT itself promotes the expression of PTEN and CD5. As a result, AKT becomes inactive (#3), thus preventing the proteasomal degradation of TP53 via mouse double minute 2 homolog (MDM2) (#4). Stabilized TP53 transcriptionally represses MYC (#5). This impedes activation of BMI1 via CITED2, and together with stabilized TP53, this now allows the transcriptional activation and nuclear localization of the cell cycle inhibitors CDKN1A/2A/2B (#6), locking the system into the anergy-matched attractor.

Next, we studied how this hypothesized regulatory circuit is disturbed during RS transformation. Our trajectory study predicted different mechanisms for each of our three simulated RS conditions, all of which led to stable states of cell cycle activation and proliferation.

In one condition, upon losses of CDKN2A/B and TP53 (Fig. 5D, top right), MYC is directly transcriptionally induced (#1), repressing the expression of CDKN1A (#2). The synergistic loss of CDKN1A and CDKN2A/B allows the activation of cyclins entering the S phase. Concomitantly, active MYC allows the stabilization of AKT via SET and protein phosphatase 2 (PP2A) (#3), oscillating activities of AKT and NOTCH1, then leading to the repression of NFAT and the exit of anergy (#4 and #5),

In the second condition of RS (Fig. 5D, bottom left), NOTCH1 gets directly activated upon AKT constitutive activation (#1), leading to a fast inhibition of NFAT (#2). AKT activity also stabilizes MDM2, thus leading to TP53 inhibition (#3), downstream reactivation of MYC (#4), and inhibition of CDKN1A (#5). In addition, MYC and AKT activities allow BMI1 stabilization, which in turn is responsible for the inhibition of CDKN2A/B (#6). Consequently, this combination leads to a stable proliferative phenotype, as described above.

In the third RS condition, upon NFAT loss (Fig. 5D, bottom right), AKT is stabilized after BCR stimulation (#1). NFAT loss also leads to the expression of S100A4, which acts redundantly with MDM2 to induce the proteasomal degradation of TP53 (#2). Downstream of these events, the trajectory leads to a proliferative attractor following the activities described for AKT hyperactivation via MYC and BMI1 activation (#3) and inhibition of cell cycle inhibitors (#4).

Together, our trajectory study highlights unknown players involved in RS in particular, SET, PP2A, and BMI1. While the relevance of these genes has been reported for CLL (*46*, *47*), their role in Richter transformation has not yet been described. Our approach thus warrants their investigation in patient cohorts and model systems.

### Driver screening: BMI1 and TP53 as players in RS transformation

**Fig. 6.**
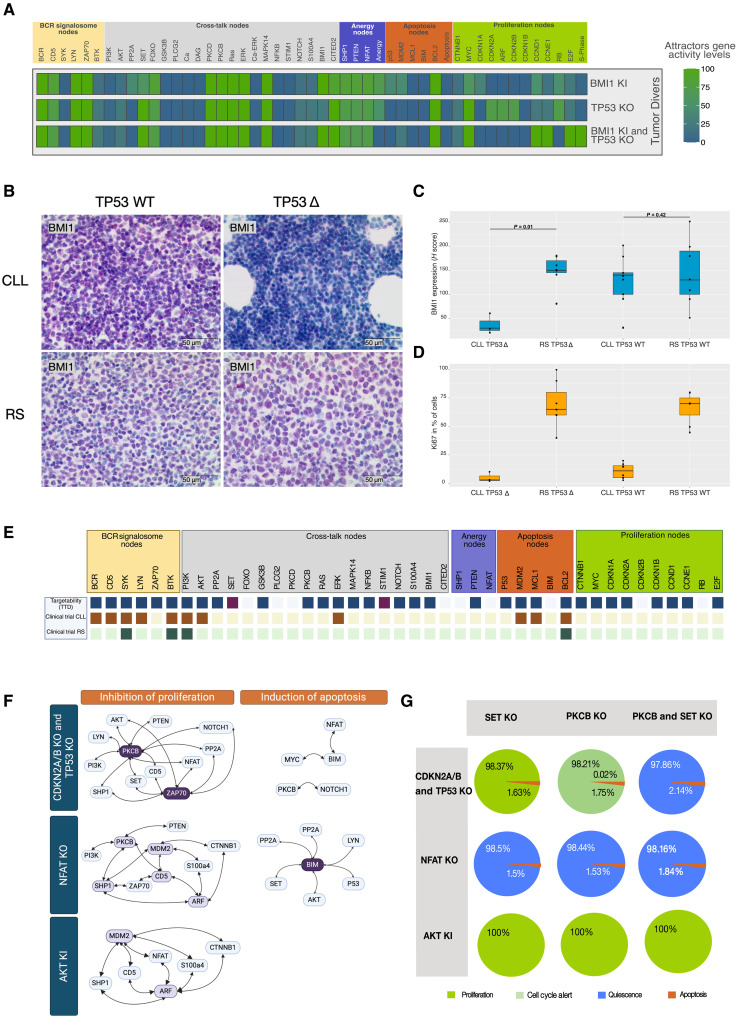
Tumor driver and drug target analyses predicted by the CLL/RS model. (**A**) Fused attractor patterns of the BMI1 KI, TP53 KO, and their combination. Attractor landscapes are fused within one state. Node activity is indicated as an averaged percentage within the attractors, weighted by their respective basin estimations. Blue indicates inactive nodes, and green indicates active ones. Nodes are grouped according to their biological process association. (**B**) Example of BMI1 immunohistochemistry stainings. (**C**) BMI1 level quantification within the TP53-mutated (∆) and WT groups of the CLL/RS cohort (*n* = 3 to 9). Boxplots depict the median and first and third quartiles. A Wilcoxon test was performed between TP53 WT and mutated cases of each entity. (**D**) Quantification of the Ki67 staining within the same four patient groups. (**E**) Visualization of the targetability of the nodes within the CLL model. Nodes are marked respectively in dark purple if targetable and in orange (CLL) or green (RS) if developed targets have reached clinical trial levels. (**F**) Visualization of the automatic perturbation screening results for three RS conditions (depicted on the left, dark blue boxes). The ability to block proliferation or induce apoptosis (on top in orange) is shown. Combined targets are depicted in the form of interactomes. A double-headed arrow indicates that two nodes have been reported as a combination. Dark purple nodes indicate targets participating in more than seven combinations, while light purple ones indicate targets participating in more than four double combinations. (**G**) Attractor landscape analysis for the combination of SET and PKCB intervention is depicted for three RS conditions (indicated by light gray boxes on the left). Attractor patterns are depicted as pie charts representing the phenotypical readout weighted by the sum of the estimated basins. Colors encoding phenotype readouts are reported in the legend.

To investigate unknown drivers of tumor evolution from CLL to RS, we conducted an automatic perturbation screening using the Java-based software ViSiBool (*48*). Here, we screened for changes in a combination of two nodes that eliminated the anergic attractor from the CLL simulations and induced a fully proliferative attractor with active S-phase instead. Considering the number of nodes in the model, a total number of nearly 5000 possible single and double hit interventions are theoretically possible. The automatic screening narrowed this number down to 300 suggested combinations. These were further manually narrowed down by selecting only exact matches with full S-phase. In addition, biologically nonmeaningful combinations were neglected. The remaining meaningful combinations are depicted in fig. S5. One of these combinations was the constitutive activation of BMI1 together with a KO of TP53. Our analysis of tumor trajectories already revealed BMI1 itself as a critical node. In addition, BMI1 is the most frequent driver selected in the screening (fig. S5). TP53 lesions are very frequent in CLL and are connected to a higher risk of transformation to RS. Thus, we further investigated whether BMI1 overactivation could serve as a “second hit” to transformation in CLL patients carrying loss-of-function *TP53* lesions. In further simulations, we found out that perturbation of either BMI1 or TP53 leads to changes in the attractor pattern compared to the unperturbed CLL, but the single lesions were not capable of inducing a fully proliferative attractor (Fig. 6A). The simulation of BMI1 constitutive activation together with TP53 KO, however, showed full induction of S-phase. To validate this finding, we performed BMI1 and antigen Kiel 67 (Ki67) immunohistochemistry staining in our CLL and RS FFPE patient cohort (Fig. 6B). In addition, we assessed *TP53* status of patients from both groups. This revealed that *TP53* mutated RS show significantly higher BMI1 expression than their CLL counterpart (*P* value = 0.01), while there was no difference in BMI1 expression within the groups of WT *TP53* (*P* value = 0.42). Also, the strong expression of BMI1 can occur in CLL but is not necessarily connected to high proliferative activity, as we detected a continuum of BMI1 expression in the low proliferating group of CLL (Fig. 6, B to D) just as a *TP53* loss alone did not necessarily lead to a more aggressive behavior of the CLL. Noteworthy that high BMI1 expression combined with a *TP53* lesion was not detected in any of the CLL patient probes, but nearly all RS probes displayed strong expression of BMI1, especially the ones harboring a *TP53* lesion.

### Drug target screening predicts personalized drug responses

After searching for tumor drivers, we also applied our model to search for drug targets. Before screening, we investigate the targetability of our nodes and the abundance of targets, as already tested in CLL and RS (Fig. 6E). For 29 nodes of the model, drugs are already developed. In addition, we identified two additional nodes via literature screening, namely, SET and stromal interaction molecule 1 (STIM1), for which experimental targeting strategies were developed (*49*–*51*). Second, we investigated how many within the targetable nodes have already reached the clinical trial phase for CLL or RS. In CLL, we found 12 targets that are currently in clinical trials. Regarding RS, only four nodes resulted in actual targets that reached clinical trials, highlighting once again the unmet medical need for clinical drug targets. To deepen our analysis, we detailed our screening by reporting the number of developed drugs, their type, and the application area (table S5). As expected, most developed compounds fall under the small molecule category. A good fraction of the identified drugs have an oncological application, highlighting their potential repurposing. In addition, to consider potential adverse effects, we reported the number of drugs that failed in clinical trials for each target.

For these reasons, we applied our model to predict potential therapeutic targets for the investigated RS conditions, again using ViSiBool (*48*). The analysis aimed to induce apoptosis or delete the proliferative attractors for each condition. Our perturbation screening exhaustively tests for up to double hits of gene/node combinations present in our network. Testing all potential combinations experimentally without filtering the simulation results would imply examination of nearly 5000 targets, which is then multiplied by each RS condition.

Our screening revealed that the CDKN2A/B, TP53 KO, and NFAT KO conditions both presented targets for inhibiting proliferation and inducing apoptosis, whereas the AKT KI condition did not present any targets for inducing apoptosis. Depending on the underlying transformation mechanism, we found different target combinations to be effective for each condition (Fig. 6F), highlighting the importance of considering the landscape of patient alterations when deciding on treatment regimens.

As an immediate example of the diversified effect of drug intervention in different conditions, we highlight the target combination of SET and PKCB (Fig. 6G). While SET and PKCB have already been targeted for the treatment of CLL in experimental studies (*49*) and clinical trials for other lymphoma entities (*52*), our in silico simulations suggest that the simultaneous targeting of SET and PKCB would lead to a fully quiescent behavior in the CDKN2A/B and TP53 KO condition but would have no effect on the AKT hyperactivation simulation (fig. S6, A to C and G). Furthermore, in our NFAT KO simulation, SET and PKCB as single KOs could already prevent proliferation, indicating no benefit from targeting both proteins simultaneously (fig. S5, D to F). To showcase transferability into actual therapeutic suggestions, we further investigated the targetability of our case study. First, we evaluated whether there indeed were already drugs available for the identified targets. PKCB is targetable by a small molecule, enzastaurin, already reaching clinical trials for multiple oncological applications (including lymphomas). Instead, a peptide (COG449) is reported for SET in the preclinical (in vitro) phase. Both drugs have a defined mechanism of action. Enzastaurin is a serine-threonine kinase inhibitor that targets PKCB together with the PI3K/AKT pathway. Notably, this off-target activity might be of interest in targeting AKT-hyperactivated RS. The drug has already been shown in preclinical data to inhibit cell proliferation and angiogenesis and induce apoptosis. COG449 acts through binding to SET, thus preventing the formation of the SET–PP2A complexes, finally inducing the activation PP2A. COG449 determined a reduction in cellular levels of the antiapoptotic Bcl-2 family member Mcl-1, overexpressed in CLL cells. In B-CLL and non-Hodgkin’s lymphomas, COG449 has been shown to increase the activity of PP2A notably and to inhibit the growth of tumor xenografts in mice. Overall, the identified drugs show promising potential repurposing applications in the context of RS.

## DISCUSSION

There are a variety of modeling strategies that can be used to investigate the dynamics of biological systems. In the presented work, we approached the problem of modeling large pathway cross-talks with a mathematical perspective by using Boolean networks. Theoretically, Boolean networks are relatively simple models, allowing qualitative simulation of a system’s dynamics. This holds in terms of time and value of the variables in the models (*53*). In contrast to Boolean networks, e.g., ordinary differential equation (ODE) systems can be used to simulate a modeled system quantitatively in terms of time and concentration values. Besides these two approaches, other types vary in their settings and resolution of the simulated dynamics. All these strategies have different benefits and drawbacks. While ODE-based models can simulate the dynamics of a system in a relatively precise quantitative fashion, their complexity is a limiting factor in model size. In addition to that, comparably much data in a good resolution are required to get considerable estimates for the underlying parameters of the model equations (*54*). Especially for biological systems, these data are expensive and often not available. In contrast, Boolean networks’ dynamics are limited to simple qualitative predictions based on binary values. The simplicity of Boolean functions compared to differential equations comes, however, with the advantage of simulating larger models. Exemplarily, Cantone and colleagues (*55*) reviewed the above-mentioned gain in size when using Boolean networks in the context of bacterial lung infection. Here, they showed that ODE models scale up to 20 nodes, whereas the scalability of Boolean models goes up to 100 nodes. Furthermore, setting up an adequate Boolean network requires less data and knowledge. Consequently, this modeling strategy is deemed more resistant to noise or uncertainty regarding parameter settings. In addition, because of the simplified logic gate approach, different regulatory levels can be represented, including epigenetic modifications when the effect on the expression of the target gene is known (fig. S1). To combine the power of both approaches, logic modeling simulations could be paired with more detailed kinetic systems to model subnetworks, as assumed adequate data are available. In addition, there are some strategies to handle the binary constraints more flexibly, like input-output analyses as used in our model (Fig. 5C) and the integration of delay nodes or so-called helper nodes. These can support a more biologically relevant time resolution within discrete models.

In general, deciding which model to use mainly depends on the research question that shall be answered. In line with the principle of Occam’s razor, we should prefer simpler models that are sufficient to provide answers to the given research question to more complex ones (*56*). Boolean models do not attempt to predict concentrations or kinetics but rather understand biological readouts at higher granularity. We decided to use a Boolean network for our qualitative analysis of the Richter transformation. Our modeled network comprises ~50 nodes, with further potential for future extensions as demonstrated in this work with the integration of TME signals.

When moving to dynamic simulations, the model was evaluated under different perturbed conditions. Considering the number of 2^49^ existing starting states of the model, we retrieved a relatively limited number of attractors, as widely described for biologically motivated networks (*57*). In addition, our simulation often resulted in fixed-point attractors, indicating the stability of the simulated network (*53*, *58*). Accordingly, we could also show that our model is stable under random perturbations. In addition, our system showed topology, dynamics, and stability properties comparable to biologically motivated models.

The phenotypes retrieved from our unperturbed CLL simulation are congruent with what is known for CLL without stimulation by the TME: a mainly anergic phenotype with low percentages of apoptosis and proliferative activity. By examining the attractor pattern on a node-wise level, we could show that the model reproduces experimental results. Our validation comprises different levels of biological regulations derived mainly from functional experimental analyses and scRNA-seq data. Besides RNA expression data, proteomics and metabolomics data are getting more and more comprehensive. It is a future perspective to implement computational procedures to use this information to validate logic models following a principle similar to our scRNA-seq validation pipeline. By adding microenvironmental factors or known high-risk lesions such as MYC activation, the received attractors changed to a more unstable pattern with active proliferation and cell cycle alert conditions but still retaining parts of the anergic phenotype as known from proliferation centers of CLL.

Also, when moving to the simulation of RS, our model was able to reproduce the behavior of published experimental RS models. Because RS is a very heterogeneous disease entity, and a plethora of different disease drivers have been found in the last few years, our different in silico RS subtypes can display the tumor heterogeneity of RS within one single disease model. For example, Kohlhaas *et al.* (*38*) demonstrated that 50% of cases showed an activation of the AKT pathway in their patient cohort. Also, it is known that activating *NOTCH1* lesions are the most common mutations in RS (*9*, *19*). In our model simulations, both the constitutive activation of AKT and NOTCH1 lead to an inactivation of NFAT, which is itself controlling the expression of CD5. This could mechanistically explain why, during CLL transformation to RS, most tumor cells show a loss of CD5 expression as observed in our model and in the CD5 expression of our FFPE patient cohort.

In our model validation using scRNA-seq data from 13 nodes examined, we observed an alignment between the mRNA data and the attractor patterns for 10 nodes. As demonstrated by the results of our input-output analysis, considering the genomic landscape of the patient cohort of Nadeu *et al.* (*21*) with mainly WT *TP53*, we can fully explain the discrepancies seen in the activity level of the three remaining nodes, namely, MYC, BMI1, and CITED2. Since in the literature, the frequency of *TP53* loss of function in RS is indicated as ~50% of RS cases (*18*, *19*), the incongruence between our model and the RNA data highlights the need to consider tumor heterogeneity when investigating molecular mechanisms of the disease.

Reflecting the deterministic nature of our model, once we validated its stable states, we could study the different trajectories leading to the RS phenotypes upon different alterations. Here, we could show that different alterations involve different circuits in the dynamic system, thus leading to alternative tumor development mechanisms. The phenomenon of mutually exclusive lesions is well known (*59*, *60*) during tumor evolution. This phenomenon covers more than one-quarter of well-known cancer genes, excluding, e.g., the co-occurrence of lesions on the same molecular pathway (*59*). Exemplarily, in a CLL and RS cohort bearing *NOTCH1* somatic mutations, it was shown that the latter was mutually exclusive with MYC lesions in cases with additional trisomy 12. Although it was demonstrated by Parry *et al.* (*17*) that *NOTCH1* and *MYC* alterations can occur in the same RS subtype, Nadeu *et al.* (*21*) described that their co-occurrence is rather rare. In accordance, NOTCH1 and MYC do belong to the same molecular trajectories also in our model, and the activation of NOTCH1 already leads to MYC activation, which could serve as a mechanistic explanation for the above-described phenomenon.

In addition to investigating the tumor trajectories, we applied the model for large-scale perturbation experiments, investigating both tumor drivers and drug targets. Considering the importance of BMI1 for CLL transformation derived from our tumor trajectory experiments, it is a confirmatory result that, in our tumor driver screening, again, BMI1 was highlighted as an important player in tumor progression. Particularly, BMI1 constitutive activity together with loss of TP53 leads to an RS-like attractor in our CLL model. Via immunohistochemistry experiments in our FFPE patient cohort, we found that CLL tumor cells show either BMI1 expression or *TP53* loss, but the combination we observed only in RS. Also, our analysis revealed that high levels of BMI1 can occur in *TP53* mutated and in *TP53* WT probes, indicating that these two genes are independently regulated. Because RS is known to be a heterogeneous disease with multiple different underlying disease drivers, we would assume that in cases with high BMI1 expression levels without *TP53* mutation, an alternative path of tumor evolution exists. Our tumor driver screening also supports this hypothesis since constitutive activity of well-known factors of CLL disease progression, such as PI3K/AKT, MAPK, or NOTCH1 activation, combined with BMI1 activity, led to a proliferative attractor.

Noteworthy, BMI1 itself is not completely unknown in the context of CLL progression. Rouhigharabaei *et al.* (*46*) showed that BMI1 can be constitutively activated via t(10;14)/IGH-BMI1 rearrangement. In their work, they examined six RS patients with *BMI1* rearrangement, one of which showed an additional subclonal loss of *TP53*. Two of their patients showed a loss of *RB*, which also negatively affects TP53 since Rb indirectly activates TP53 via inhibition of MDM2 (*61*). This finding, again, confirms the hypothesis of a synergistic effect of BMI1 and TP53. Last, one patient showed trisomy 12, which is in 40% of cases connected to activating *NOTCH1* mutations (*62*). Here, BMI1 activation possibly acts synergistically with NOTCH1, a combination which, as described above, also leads to a proliferative attractor in our model.

From a general perspective, our drug target and tumor driver screening appear as two faces of the same coin. Our analysis of available drugs in development supports the transferability of our approach to a real case scenario. Exemplarily, our evaluation on BMI1 as a tumor driver could not only serve a diagnostic purpose but also represent an actionable target for *TP53*-mutated CLLs that have the potential of transformation to RS. On the other hand, the personalized aspect of our drug screening approach, investigating targets for different underlying molecular alterations, could be used to develop and experimentally test biomarkers for drug responses.

In this direction, these concepts are already considered, e.g., in the contexts of molecular tumor boards, where the genetic alteration landscapes of single patients are considered when deciding over oncological treatments, and repurposing approved treatments for different neoplasia with similar alterations.

In the current CLL model, the main focus lies on highly studied oncogenic signaling pathways. Boolean Network comes with the acquired advantage of considerable adaptability in integrating experimental evidence. In the past few years, it has been shown that CLL shows high metabolic plasticity while shuffling between the different TMEs in peripheral blood, bone marrow, and lymph nodes. Specifically, CLL performs a glycolytic switch when entering the lymph node from the peripheral blood with high levels of glycolysis and predominantly glutaminolysis to fuel the tricarboxylate cycle and mitochondrial oxidative phosphorylation (OXPHOS) (*63*, *64*). This metabolic reprogramming might be stimulated via active NOTCH1-MYC axis, triggered via BCR and CD40 signaling by the TME (*63*, *65*). After transformation to RS, although the mechanism to activate OXPHOS metabolism seems to be differentially regulated since recently, Nadeu *et al.* (*66*) elucidated an RS phenotype with high OXPHOS but low BCR signaling, possibly via activation of mammalian target of rapamycin (mTOR)/MYC pathway. However, the detailed understanding of how oncogenic signaling is connected to metabolic adaptation in CLL and RS is still incomplete.

Nevertheless, with more knowledge emerging from this field, one future approach could be integrating different TME influences and the connected metabolic conditions to our CLL model to simulate changes in the CLL metabolic network activities during the shift through different anatomical localizations. Because there is also strong evidence that metabolic reprogramming is connected to Venetoclax drug resistance (*64*), it would be a promising approach to integrate these upcoming metabolic pathways to drug target screenings and also to investigate mechanistic insights of altered drug sensitivity depending on the metabolic status of the leukemic cell. In general, drug target screenings via a Boolean network model, as shown here, can guide experimental testing of unknown drug combinations. This approach can potentially reveal drug synergies that would not be expected from the classical point of view. Predictions from automatic drug target screenings should, in a next step, be challenged by experimental and clinical studies.

Our work presents a CLL model to the community, combining a systems biology approach with experimental readouts. Our model can be used in synergy with experimental systems to either integrate upcoming information into the model or to guide wet-laboratory approaches, aiming for a mutual enrichment of the different model systems and lastly for a deeper understanding of the complex process of Richter transformation.

## MATERIALS AND METHODS

### Boolean network models: Formalism and simulation strategy

Boolean network models are characterized by a set *X* = {*x*_1_, …, *x_n_*} of variables *x_i_*,*i* ∈ {1, …, *n*} that represent n genes or proteins (*53*, *67*). Each variable *x*_1_ can have either a positive (active, 1) or zero (inactive, 0) value *x_i_* ∈ {0,1}. Regulatory interactions within the model are summarized in a set *F* = {*f*_1_, …, *f_n_*} of Boolean functions. Each function *f*_i_,*i* ∈ {1, …, *n*} is constructed via logical operators [AND (&, ∧), OR (|, ∨), and NOT (!, −)]. The assignment of each variable at one point in time is determined on the basis of applying the given Boolean functions on the respective assignments at the previous time step (*67*). The state of a Boolean network at a certain point in time *t* is defined by a vector $\vec{x\left(t\right)}=\left[x_{1}\left(t\right),\ldots ,x_{n}\left(t\right)\right]$ . Considering all combinations of value assignments to the *n* variables, the total number of states in the network sums to 2*^n^* possible states. Based on three different updating strategies, the transition from one state to its successor is computed. Namely, these are synchronous, asynchronous, and probabilistic Boolean network models. In synchronous Boolean network models used in this work, all Boolean functions are updated simultaneously at each discrete step in time *t*. Under a synchronous update scheme, Boolean network models eventually converge on recurring sequences of states, called attractors. Depending on the sequence of recurrent states, there are single-state (fixed points) and multiple-state (cyclic) attractors (*53*). All states leading to a particular attractor define its so-called basin of attraction. The larger this basin of attraction is, the higher the probability that an attractor will be relevant from a biological perspective (*68*). From an interpretative perspective, attractors describe the long-term behavior of the model and are associated with biological phenotypes. This is crucial for the iterative modeling process, allowing attractors to be validated and compared to experimental results and lastly allowing the network to be refined. Experimental KI/KO can then be performed in the system by fixing the value of a node to 0 or 1 throughout the model simulation.

Considering the 49 nodes of the CLL model, the complete state space would count 2^49^ states, prohibiting exhaustive simulations. To circumvent this issue, we applied an approximation algorithm called SAT algorithm (SAT standing for satisfiability), implemented in BoolNet, which exhaustively retrieves the attractors of the system. It is approximate, since the SAT algorithm does not compute the basins of attraction, we therefore estimated the basin sizes based on 10 million randomly drawn starting states under a synchronous update scheme. This strategy was applied for the attractor simulations of the unperturbed CLL model and the RS conditions and the TME simulations.

**Fig. 7.**
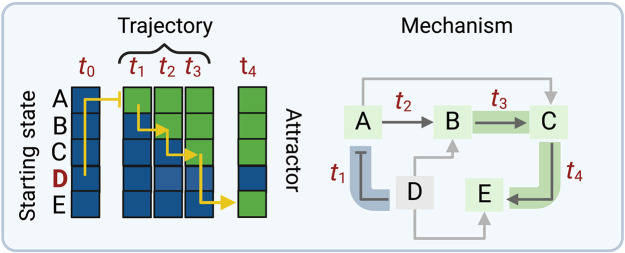
Principle of trajectory studies. The figure depicts the principle of trajectory studies, which are applied to investigate the different mechanisms leading to CLL and RS attractors. Starting from an initial state *t*_0,_ the model is simulated under a synchronous update scheme (left side). Here, time steps up to the attractor (*t*_4_) are depicted. Considering the logical dependencies between nodes in the model, the main regulatory dependencies involved in each time step are highlighted in the form of yellow arrows on the trajectory. For visualization purposes, the main regulatory dependencies are depicted as highlighted arrows (green if regulation is active, blue if regulation is inactive) on an interaction graph representation (right side). Time points where the regulatory dependencies occur are depicted in dark red, both in the attractor trajectory and the interaction graph representation.

Besides studying the system’s long-term behavior, it is also possible to investigate the trajectories leading to attractors. Under a synchronous update scheme, once a starting state is defined, a deterministic sequence of states will lead to the corresponding attractor. The trajectory study is applied to study the regulatory mechanisms governing the system’s behavior (*53*, *69*). In our study, we computed trajectories starting from all nodes set to zero except for the node BCR, which is set to 1. This is because our system has a biologically motivated dependency on BCR receptor activity. Trajectories were studied in unperturbed (CLL) and perturbed conditions (our RS conditions). Here, it is possible to deduce the primary circuits activated by the different simulations investigating the node activity changes between one discrete time point and the following one. The trajectory study is performed by considering the logical connections within the Boolean functions for each node. The principle of the trajectory study is depicted in Fig. 7.

Simulations were performed using the R-package BoolNet (*70*) (version 2.1.7).

### CLL model establishment and interpretation of attractors

The modeling strategy adopted in our work follows the red line given in Ikonomi *et al.* (*69*). For constructing the Boolean network model describing CLL cell behavior, we performed extensive literature research collecting published papers from NCBI, Google Scholar, and Metacore (Clarivate). The present work prioritized studies from mouse models, human peripheral blood CLL cells, and CLL cell lines or non-neoplastic B cells when available. This brought to the evaluation of 228 published experimental works (table S1). The experimental results found in these papers provided the grounds for constructing one Boolean function for each node of our CLL network. For all components of the model, different levels of regulation have been considered, e.g. cooperation of proteins (AND gates), independent activators (OR gates), or proteins inhibitors (NOT gates). The different regulatory levels were joined within the Boolean logic via AND gates (fig. S1). Detailed descriptions of all interactions and resulting Boolean network functions can be found in table S1. As an extension of the model, we also introduce microenvironmental signals encoded in the node TME in our analyses. The node is introduced as an input node that similarly activates the nodes AKT and NF-κB (tables S2 and S3).

To ease the interpretation of model simulations, we built a series of indicator nodes—namely, anergy, proliferation, and apoptosis—that were used for the interpretation of the attractor patterns in terms of phenotypical outcomes. In our model, anergy is encoded by the presence of NFAT, together with the inactivity of NF-κB, AKT, and the absence of S phase (Fig. 2G). Cells in a proliferative state are assumed to be regulated by the activity of the S phase node, which is regulated by the concomitant presence of E2F transcription factor (E2F) and cyclin E (CCNE1). Apoptosis is encoded by the concomitant presence/activity of Bcl2 interacting mediator of cell death (BIM) and by inactivity of AKT. We considered our attractors to be in a quiescent state when anergy, S phase, Cyclins, and apoptosis are all inactive [gap 0 (G0) state]. Cell cycle alert, instead, was assigned to attractors with cyclin D (CCND1) and CCNE1 activity, without entry into the S phase.

**Fig. 8.**
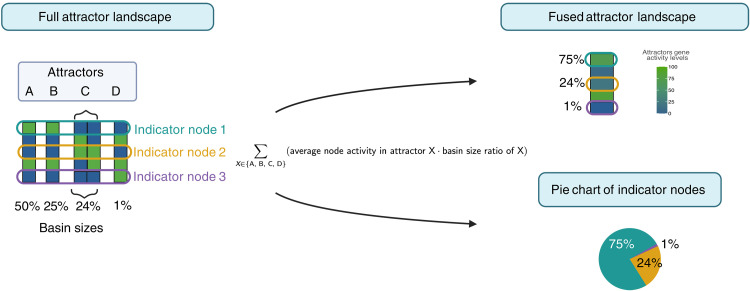
Attractor landscape representations. Attractor landscapes are represented in three different ways. First, the full attractor landscape is represented (green = ON, blue = OFF). Each attractor pattern is visualized with the corresponding estimated basin size, including the indicator nodes. Second, the fused attractor landscapes summarize all attractors for a specific simulation condition in one heatmap for each node. Here, each node’s activity is summarized as the average activity of the nodes multiplied by the basin size ratio. This visualization supports the comparison of different conditions. Finally, attractor landscapes can be represented through their indicator nodes. In the pie charts, each indicator node has a color assigned, and the size in the pie chart indicates the total covered estimated basin size. This last representation supports a concise comparison of the phenotypical interpretation of the attractor landscapes.

For visualization, attractor landscapes are presented either fully or collapsed to single states as heatmaps, as presented also by Park and colleagues (*71*) (Fig. 8). In the latter case, the activity of each gene in the final collapsed attractor is averaged based on the gene’s activity in each attractor resulting from the simulation, multiplied by its basin size ratio. Full attractor landscapes (for each node and its corresponding estimated basin of attraction) are provided for each simulation in the Supplementary Materials (fig. S2, A to H). Last, attractor landscapes are also encoded in pie charts, where attractors are represented in slices grouped by the activity of the indicator nodes (anergy, apoptosis, and S phase) and weighted by the total estimated basin sizes as a percentage (*72*).

### Model stability assessment and scale freeness

To evaluate the resistance to random noise of our CLL model, we assessed its stability toward random perturbations. We compared the resistance to noise of our model compared to randomly generated models with similar topological properties in terms of number of nodes and regulations per node. The analysis procedure is included in the package BoolNet (*70*) (version 2.1.7). Specifically, 1000 random networks were generated using the function “TestNetworkProperties()” that calls the “GenerateRandomNKNetwork()” present in BoolNet. The parameters *n* and *k* refer to the number of nodes in the network (*n*) and the number of inputs of a given node (*k*). The parameters are chosen accordingly to the values present in the original CLL model. To apply noise, we use the widely applied strategy of bitflip perturbations, where the assignments of randomly selected nodes are toggled from 1 to 0 or vice versa (*73*). Once a bitflip is applied to a network state, the corresponding successor state for both the perturbed and unperturbed states is calculated. The difference between the two successor states is calculated using the normalized Hamming distance, which measures the number of differing bits between two state vectors. Formally, it can be described as

$$H\left(x,y\right)=\sum_{i}^{n}x_{i}⊕y_{i},\forall x,y\in B^{n}$$

The normalization is performed by dividing the measured Hamming distance by the number of bits in the vectors

$$\mathrm{HD}\left(x,y\right)=\frac{H\left(x,y\right)}{n},\forall x,y\in B^{n}$$

Notably, a Hamming distance of 0 indicates that the applied perturbation had no effect on the network behavior, and the two states *x* and *y* are identical.

Scale freeness is another important property of biologically motivated networks. If a Boolean network shows a scale-free network topology, then it can be described by the power law distribution, *P*(*k*) ∝ *k*^−α^, where α is the power law scaling parameter and *k* is the number of connections in the network.

### Model validation: scRNA-seq pipeline

We used a published single-cell RNA dataset of human CLL patients (*21*) to validate our model’s hypothesis. The dataset comprises longitudinal samples from three patients, taking the samples at different time points of the disease. For our analysis, we took the published Seurat objects and selected all patients, including Richter-positive samples, according to the given metadata (ID: 12,9,365). For each patient, we extracted the genes matching the gene set in the modeled Boolean network and markers to identify Richter-positive and CLL cell samples in the data.

In single-cell data analysis, sparsity presents a substantial analytical challenge, particularly due to the prevalence of missing or zero expression values across measured cells (*74*). To address this, we deliberately selected an algorithm designed to be robust against missing data: the BASC-A algorithm, implemented in the R package BiTriNa (version 1.3.1) (*75*). We applied BASC-A to binarize the data for each patient and gene individually. The BASC-A algorithm operates by sorting expression values in ascending order and fitting step functions to the data, allowing it to detect significant discontinuities that serve as potential thresholds. Unlike traditional mean-based thresholding approaches, BASC-A is inherently resilient to sparsity and unaffected by the number of zeros in the dataset. At each identified discontinuity, the algorithm computes an approximation error, and both the threshold position and approximation error are jointly optimized through a scoring function to ensure a robust binarization outcome (*76*). To maintain the quality and interpretability of the binarized dataset, we excluded genes that exhibited no expression across all cells and genes that did not meet the significance criteria for binarization based on the statistical test embedded within the BASC-A framework.

We further selected the binarized data of each sample for proliferative cells according to their binary signature of the gene sets CCNE1, CCNE2, CCND1, CCND2, and CCND3. Each cell with at least one of these genes set to 1 in the binarized data was labeled as RS.

After selection, the data of all Richter-labeled cells were integrated across the three donors individually. For comparison to attractors, nodes mostly regulated at the expression level were subselected, namely, CD5, SET, BMI1, CITED2, NOTCH1-4, S100A4, PTEN, NFATC1, NFATC2, TP53, MYC, CDKN1A, CDKN2A, CDKN2B, and CDKN1B. Comparison to attractors of the modeled network was performed by counting the number of overlapping gene signatures for each cell. Over the different RS simulated conditions, attractors with full proliferative traits were taken for comparison, in accordance also to the selection of the single cells.

UMAP plots were created using the R-package Seurat (version 5.0.0) (*77*). To remove batch effects between the different samples, the Harmony algorithm (*78*) was applied (Harmony R-package, version 1.1.0)

### Patient cohort/FFPE tissue bank

We retrospectively analyzed FFPE samples from 28 patients with CLL and 23 patients with RS. Each patient’s TP53 status (WT, TP53 mutation, or chr 17p deletion) was assessed, if available, from routine diagnostic procedures. The samples were used by consent under approval from the local ethics committee (Ulm Ethics Committee, vote 64/24) and in conformity with the Declaration of Helsinki.

### Immunohistochemistry

Immunohistochemistry was performed on 3-μm FFPE tissue sections. The following antibodies were used: mouse anti-CD20 (1:500; clone L26, Dako, Germany), mouse anti-CD5 (1:100; clone 4C7, Dako), mouse anti-KI67 (1:200; MIB1, Dako), and rabbit anti-BMI1 (1:200; D20B7, Cell Signaling Technology).

The RED Kit (K5005, Dako) was used to detect antigen-bound antibodies. Counterstaining of the cell nuclei was achieved by staining with hematoxylin for 5 min and incubating in H_2_O for 10 min at 20°C.

Two independent pathologists did quantitative evaluation of immunohistochemistry stainings according to the *H* score. The intensities of the immunohistochemistry stainings were binarized by a threshold defined by a receiver operating characteristic (ROC) curve using the R-package pROC (version 1.18.0) maximizing the assignment to the two experimental conditions. According to this threshold, expression levels above the threshold were considered positive, and expression levels below the threshold were considered negative. We measured the difference between these two groups. In addition, BM1 expression was compared between the TP53-mutated and the TP53-WT group.

### Input-output analysis

The binary activities inherited from using the Boolean logics were extended by studying input-output relationships. This analysis was implemented and performed as described primarily by Helikar *et al.* (*79*) and further reutilized by Park *et al.* (*36*). However, in our specific case, we extended the previous work to the analysis of two inputs and visualized the resulting output activities in the form of a heatmap. The general procedure followed in the analysis is summarized below. The activity level of an output node of interest is defined as the ratio of ON (*1*) states throughout a preselected number of time steps in which the simulation is performed. The activity of an input node within the same simulation is set to a discrete percentage (from 1 to 100%) of ON states throughout the simulated time steps. For two input nodes, every combination of input starting activity percentages is thus simulated. The starting states of the noninput nodes in the network are set randomly and then synchronously updated over the number of selected time steps.

In our specific case, we performed the input-output analysis by studying as inputs “NOTCH1” and “TP53” and recording as output all the rest of the nodes in the model. For each percentage combination of inputs from 1 to 100% (one dot in the heatmap), we averaged the simulation results from 250 randomly drawn starting states. For each starting state in each percentage combination of inputs, 500 time steps were simulated, and the output results were calculated on the basis of the mean activity over the last 100 time steps.

### Automatic drug target and tumor driver screening

Laboratory screening of promising therapeutic interventions is cost-intensive and can be time-consuming. Therefore, automatic in silico screenings based on the dynamic perturbation nodes within Boolean network models can help to identify unknown intervention targets. We used our developed Java application, ViSiBool, to screen for meaningful intervention targets and tumor drivers in the established Boolean network model (*48*). This automatic screening approach allows selecting which attractor(s) should or should not be present in the final perturbed system and the expected final desired behavior of nodes of interest in the network. In addition, it is possible to set the number of intervention combinations *m* for a network of size *c*. Based on these specifications, the attractors performing all possible intervention combinations are computed in parallel [in total $\sum_{i=1}^{m}\left({}_{i}^{c}\right)\cdot 2^{i}$ ], and a list of interventions that show the preindicated long-term behavior is returned (*48*). The user can also specify preselected desired activities of single nodes that should be altered in the perturbed system.

For the drug targets, we investigated single and double hit interventions under different initial RS mutated conditions. We selected the proliferation-related attractor as the one to be deleted by the system and focused on changing the activities of the “proliferation” and “apoptosis” nodes. For the tumor drivers screening, we investigated combinations of one- and two-hit interventions that were able to induce a full activation of S phase.

### Screening for druggable targets in the CLL model

The in silico identified drug targets were compared to already developed drugs. The druggability of the CLL network nodes was evaluated by screening the Therapeutic Target Database (TTD) and the Clinical.trials.gov database. All nodes of the CLL model have been evaluated for the screening, except for indicator nodes and nodes representing chemicals (e.g., Ca^2+^) or particular activation states of a specific protein (e.g., calcium ERK). These nodes are automatically assigned as “not targetable” for both screenings. The TTD screening focused on whether each node of interest is targetable by any developed drug. The results from TTD were further integrated by a manual literature search. The Clinical.trials.gov screening focused, instead, on investigating not only whether the nodes included in the model are druggable but also whether the developed drug has reached clinical trial specifically for CLL or RS.

### Statistical analysis

We calculated the robustness of the modeled Boolean network using computer-intensive testing. To quantify the robustness, we created random Boolean networks of similar size as the modeled CLL network and used the described stability assessment. To do so, we repeatedly drew 1000 starting states at random. Randomly drawn starting states for both the original CLL network and each of 1000 random networks generated as described above. To determine if the robustness of the modeled network is increased compared to the given random networks from a statistical point of view, we computed *P* values by counting the number percentage of cases where the perturbation in the modeled network leads to a smaller Hamming distance then in the random networks. A *P* value below 0.05 is considered to be significant.

To test whether our CLL model follows a scale-free architecture, we tested whether the power law distribution plausibly describes the degree distribution of the network (*P* > 0.1) by using the R-package poweRlaw (version 0.70.6) (*80*).

For the analysis of the immunohistochemistry experiments, we used 23 samples from patients with RS and 28 from patients with CLL. The significance of the difference between the two groups was assessed by a two-sided Wilcoxon rank sum test.

BMI1 expression in the groups of TP53-mutated and TP53 WT disease entities were compared using two-sided Wilcoxon rank sum test. For both tests, we applied a significance level of 0.05.
