# Supplementary material for: CLL to Richter syndrome: Integrating network strategies with experiments elucidating disease drivers and personalized therapies
Source: Sci Adv. 2025 Sep 12;11(37):eadu7705. doi: 10.1126/sciadv.adu7705 (PMC12428933; doi:10.1126/sciadv.adu7705)
Supplement: Supplementary file 1 — Figs. S1 to S7 Tables S1 to S5 [file sciadv.adu7705_sm.pdf]

Science Advances
AAAS

Supplementary Materials for

CLL to Richter syndrome: Integrating network strategies with experiments elucidating disease drivers and personalized therapies

Julia Maier *et al.*

Corresponding author: Hans A. Kestler, hans.kestler@uni-ulm.de

Sci. Adv. **11**, eadu7705 (2025)
DOI: 10.1126/sciadv.adu7705

This PDF file includes:

Figs. S1 to S7
Tables S1 to S5

Supplementary Figures

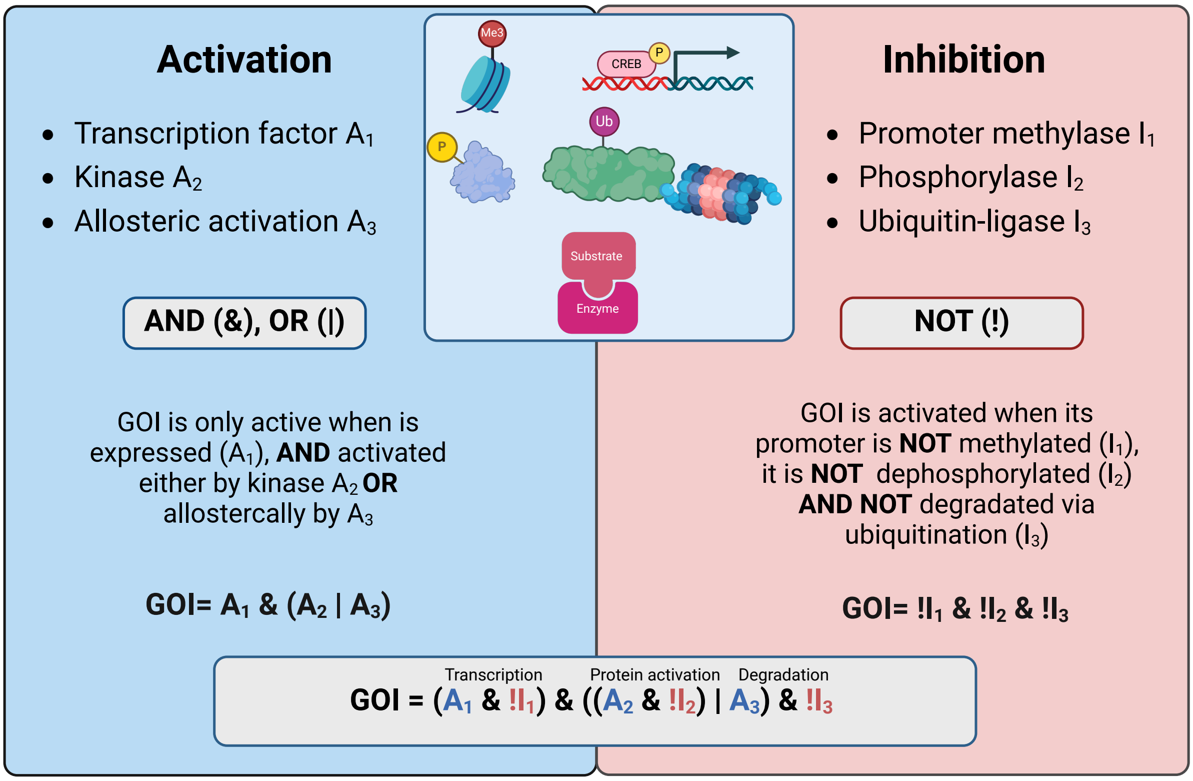

Figure S1: Construction of Boolean functions from experimental evidence demonstrated for a fictive GOI. Boolean functions for a gene \node in a model summarize all regulatory dependencies for which experimental evidence is present. In general, regulations are divided in activations (left side in blue) and inhibitions (right side in red). Activators (A) can be connected either by OR (|, $\vee$) or AND (&, $\wedge$) gates. And example for each of those is reported in the figures. In general, AND gate represent cooperative interaction where both partners need to be present in order to activate the target Gene of Interest (GOI). Inhibitors (I) instead are encoded via the NOT (!, $\neg$) gate. Generally, inhibitors are considered independent and connected via AND gates. Again, an example on the translation of published regulations into rule is presented in the figure. Finally, inhibitors and activators are combined to generate the final Boolean function (light grey box below). To combine the different levels of regulations, the subparts of the boolean rules are connected via AND gates. That implies that the GOI will be finally active only if transcriptional (referred as transcription), and post transcriptional (referred as protein activation and degradation) regulations are properly activated/inhibited and if degradation of the protein is inactive.

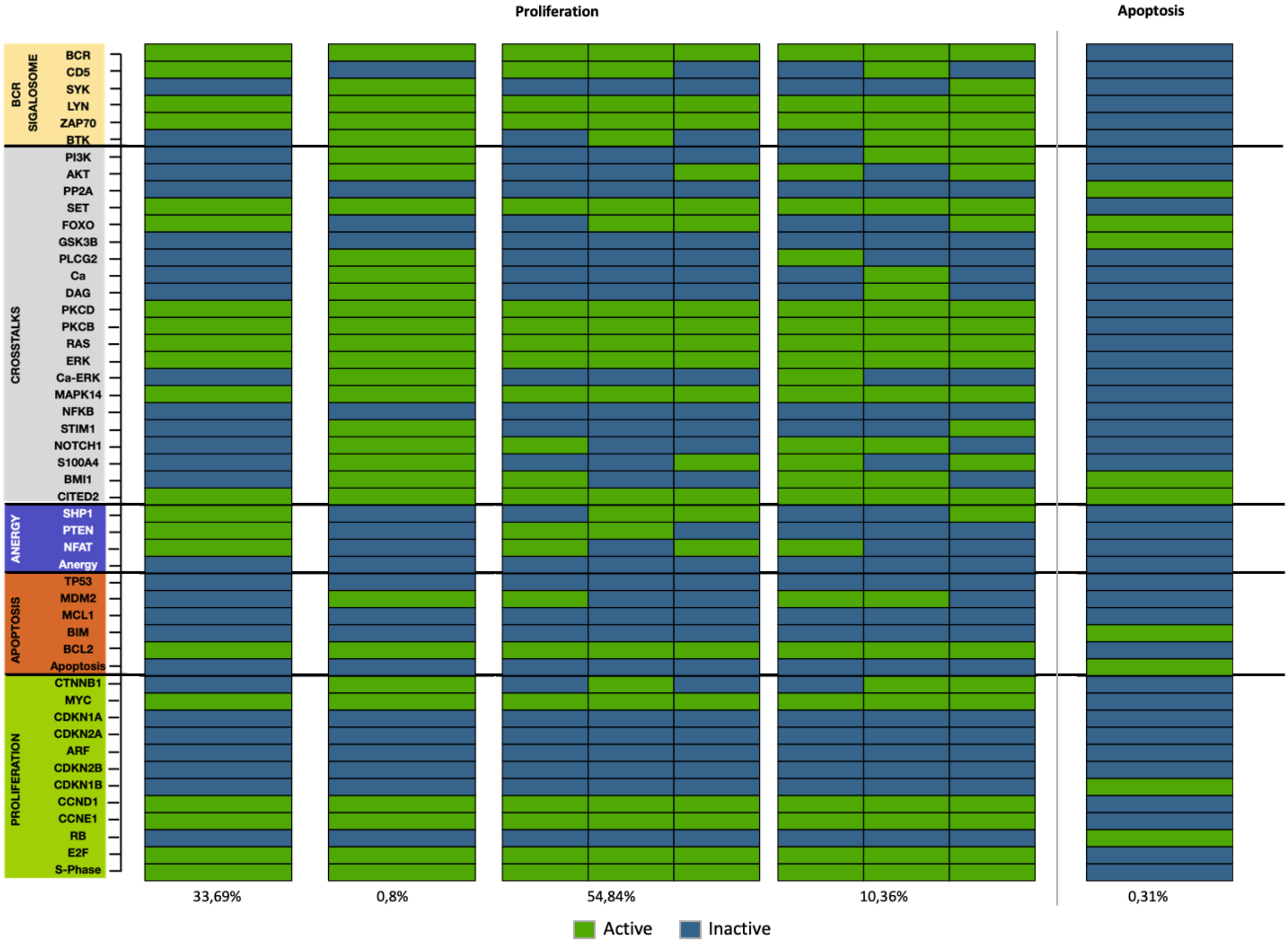

Figure S2a: Attractor pattern of the CLL model with in silico knock-out of CDKN2A/B and TP53. Two single state attractors and two cycling attractors with three states each representing proliferating cells and one single state attractor showing induction of apoptosis. The network components are listed on the left, while the state of each protein is represented by green (=active) and blue (=inactive) rectangles. Percentages below the attractor pattern shows the size of the basin of each attractor.

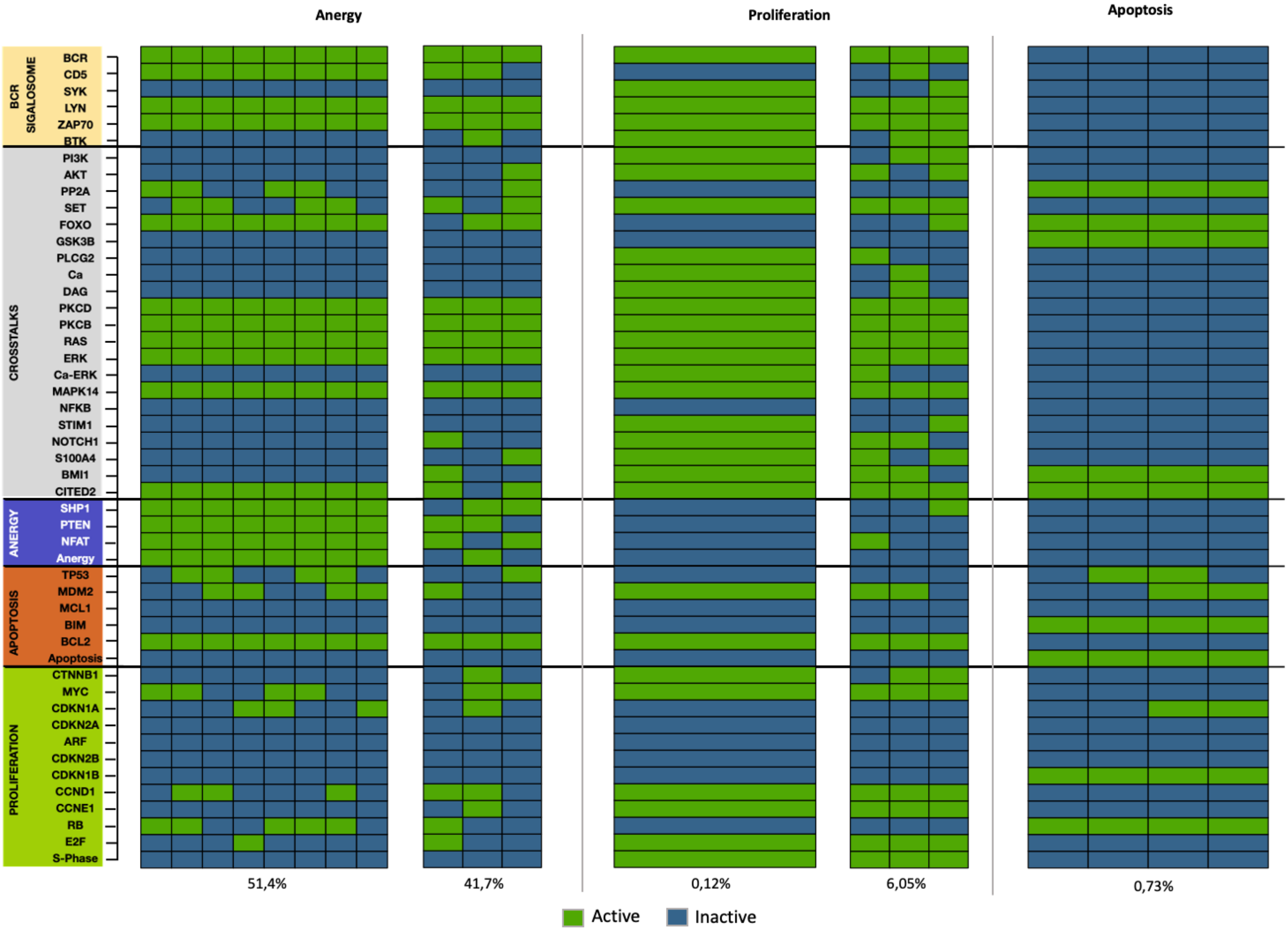

Figure S2b: Attractor pattern of the CLL model with in-silico knock-out of CDKN2A/B only. Two cycling attractors with three states and eight states representing cells in an anergic state, one single state attractor and one cycling attractor with three states represent a proliferating phenotype and one cycling attractor with four states showing induction of apoptosis. The network components are listed on the left, while the state of each protein is represented by green (=active) and blue (=inactive) rectangles. Percentages below the attractor pattern shows the size of the basin of each attractor.

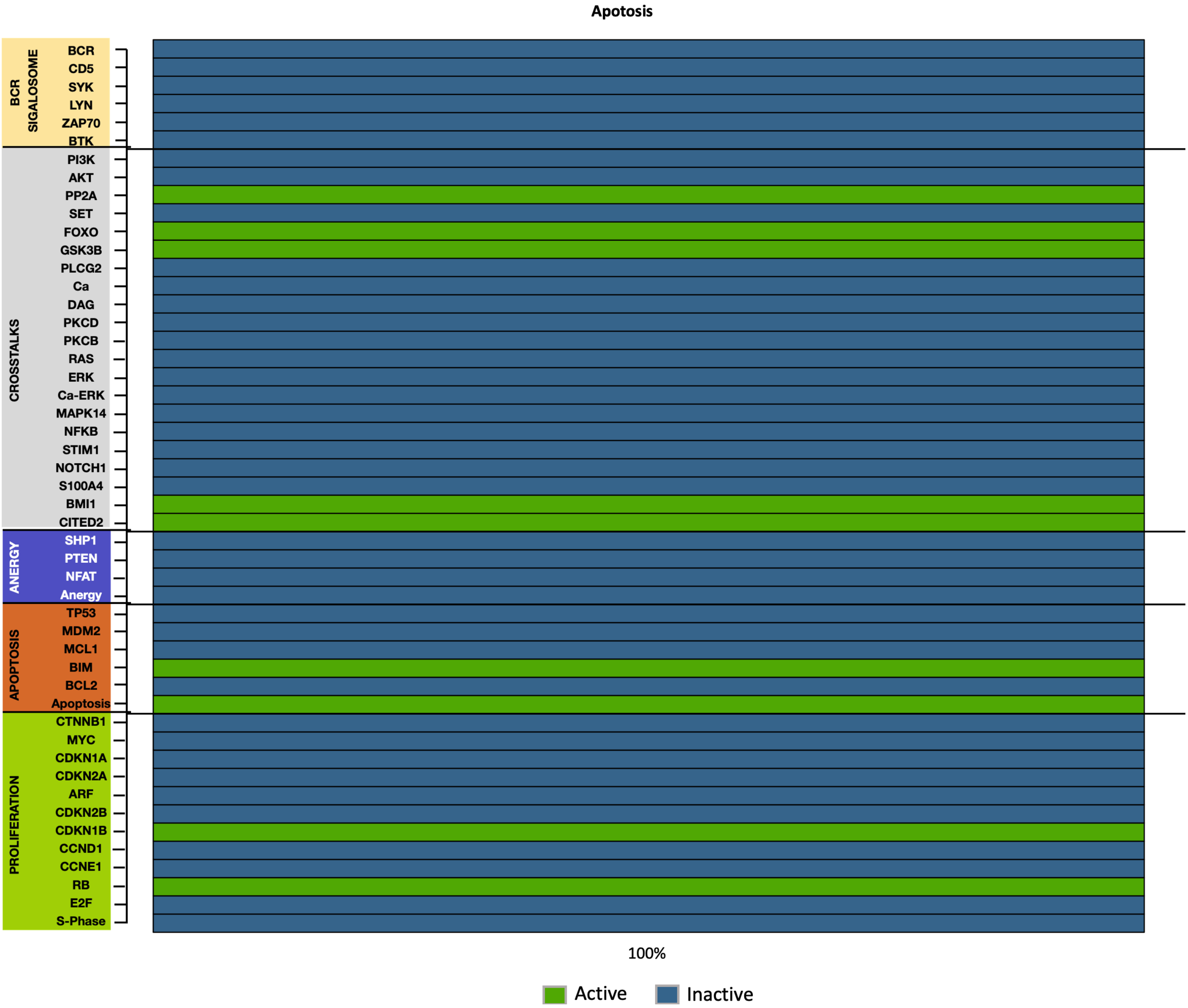

Figure S2c: Attractor pattern of the CLL model with in-silico knock-out of CDKN2A/B, TP53 and BCR. It shows one single state attractor with induction of apoptosis. The network components are listed on the left, while the state of each protein is represented by green (=active) and blue (=inactive) rectangles.

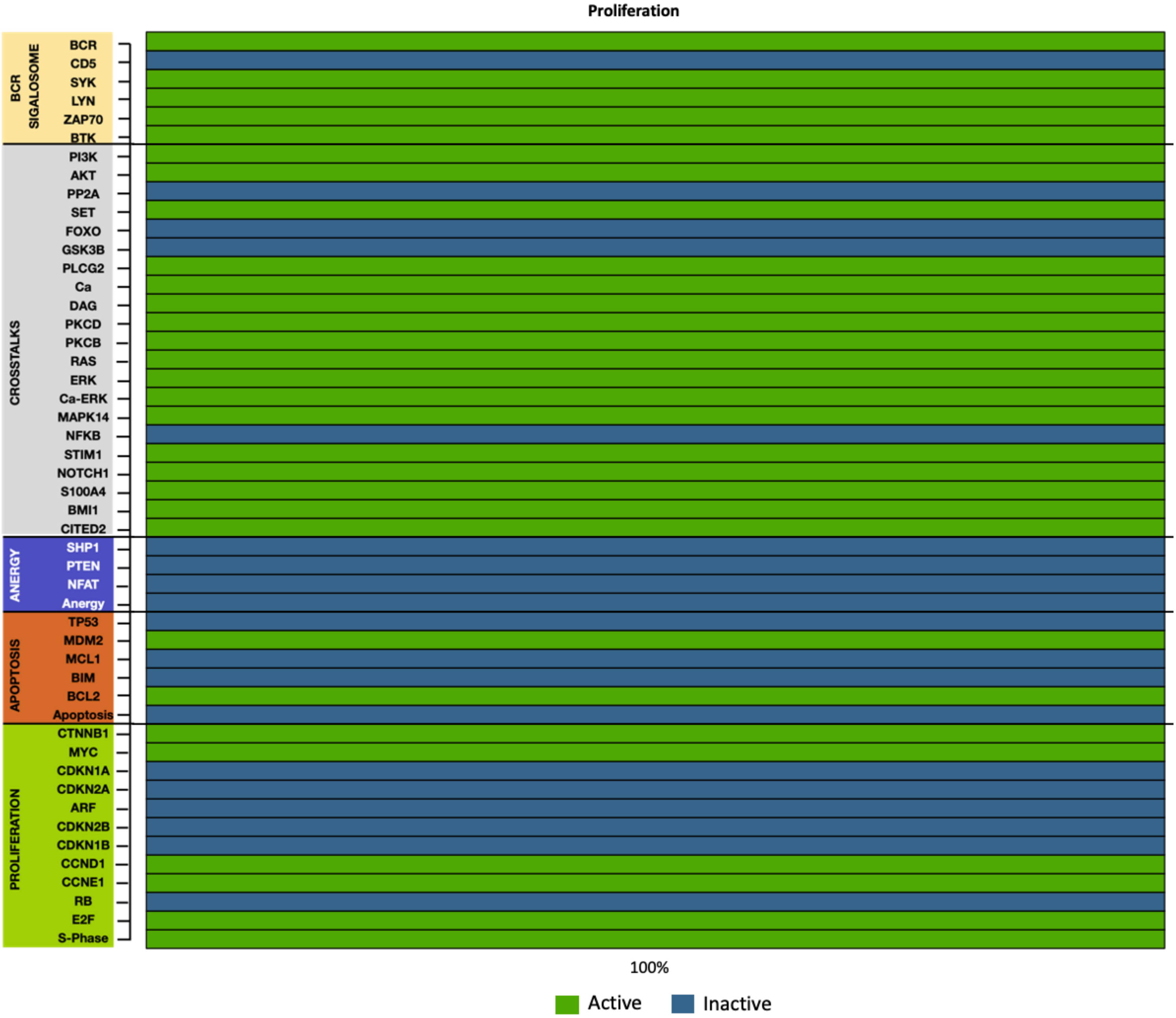

Figure S2d: Attractor pattern of the CLL model with in-silico knock-in of AKT. It shows one single state attractor representing proliferating cells. The network components are listed on the left, while the state of each protein is represented by green (=active) and blue (=inactive) rectangles.

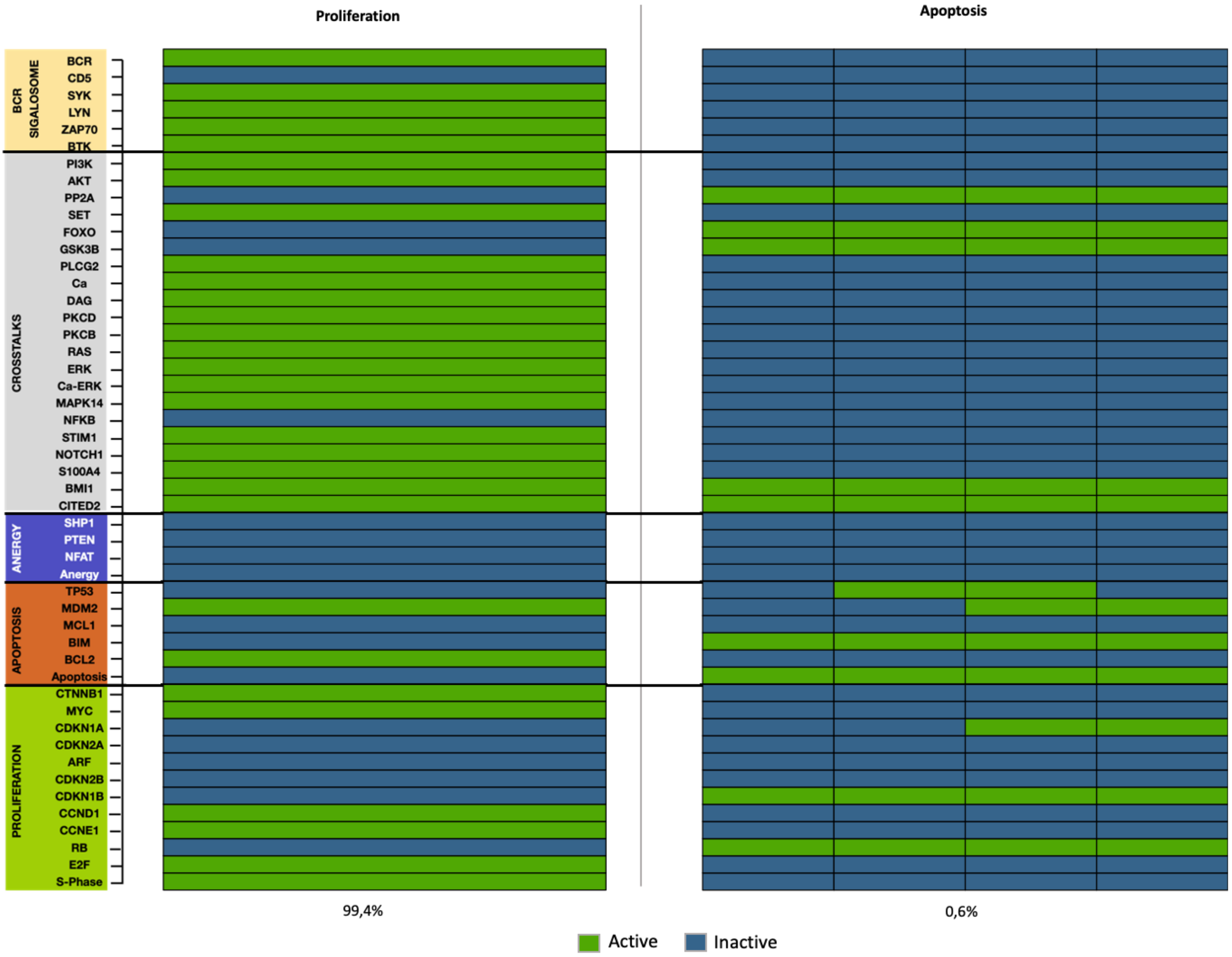

Figure S2e: Attractor pattern of the CLL model with in-silico knock-out of NFAT. It shows one single state attractor representing proliferating cells and one cycling attractor with four states showing induction of apoptosis. The network components are listed on the left, while the state of each protein is represented by green (=active) and blue (=inactive) rectangles. Percentages below the attractor pattern shows the size of the basin of each attractor.

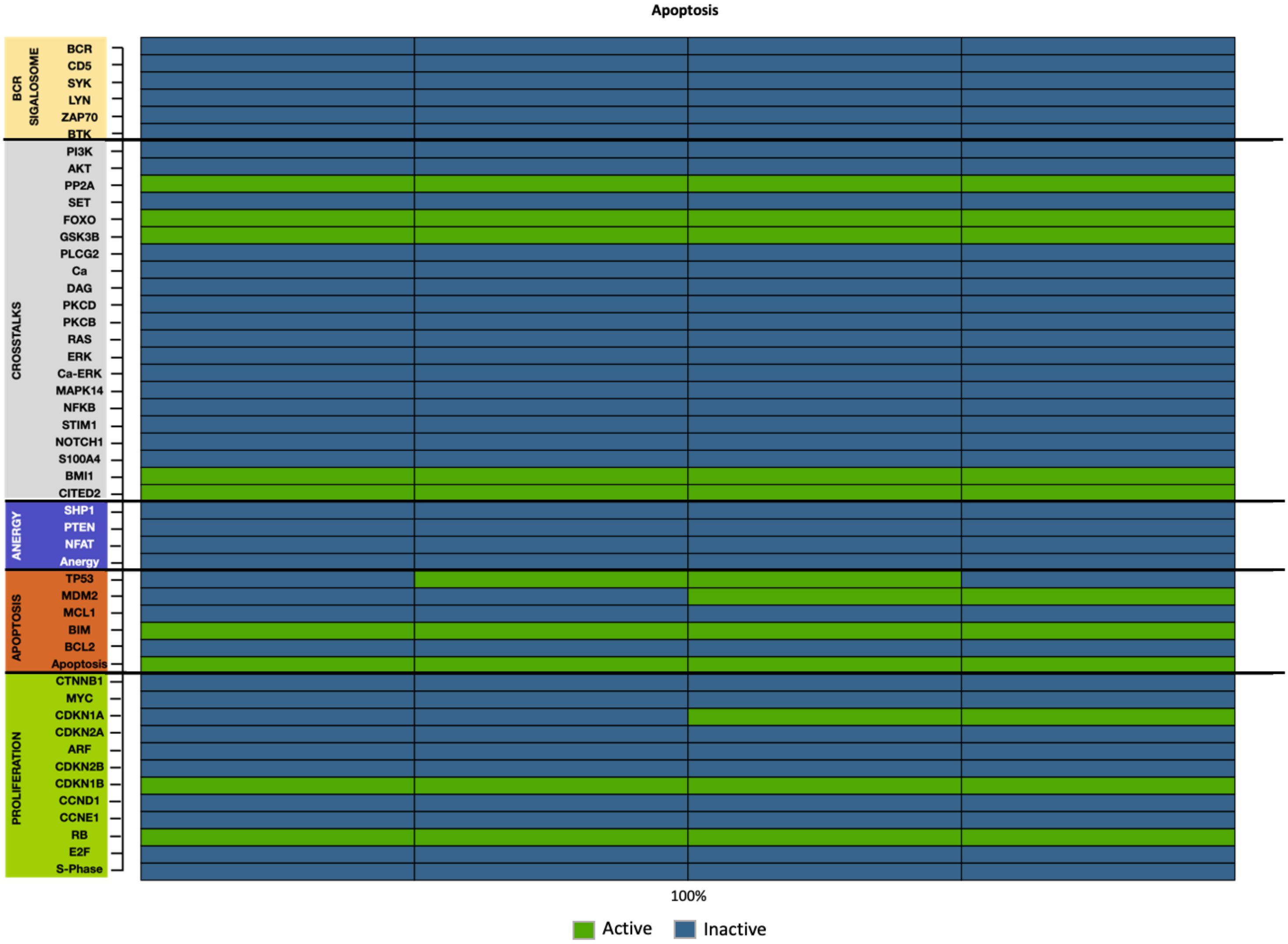

Figure S2f: Attractor pattern of the CLL model with in-silico knock-out of BCR. It shows one cycling attractor with four states showing induction of apoptosis. The network components are listed on the left, while the state of each protein is represented by green (=active) and blue (=inactive) rectangles. Percentages below the attractor pattern shows the size of the basin of each attractor.

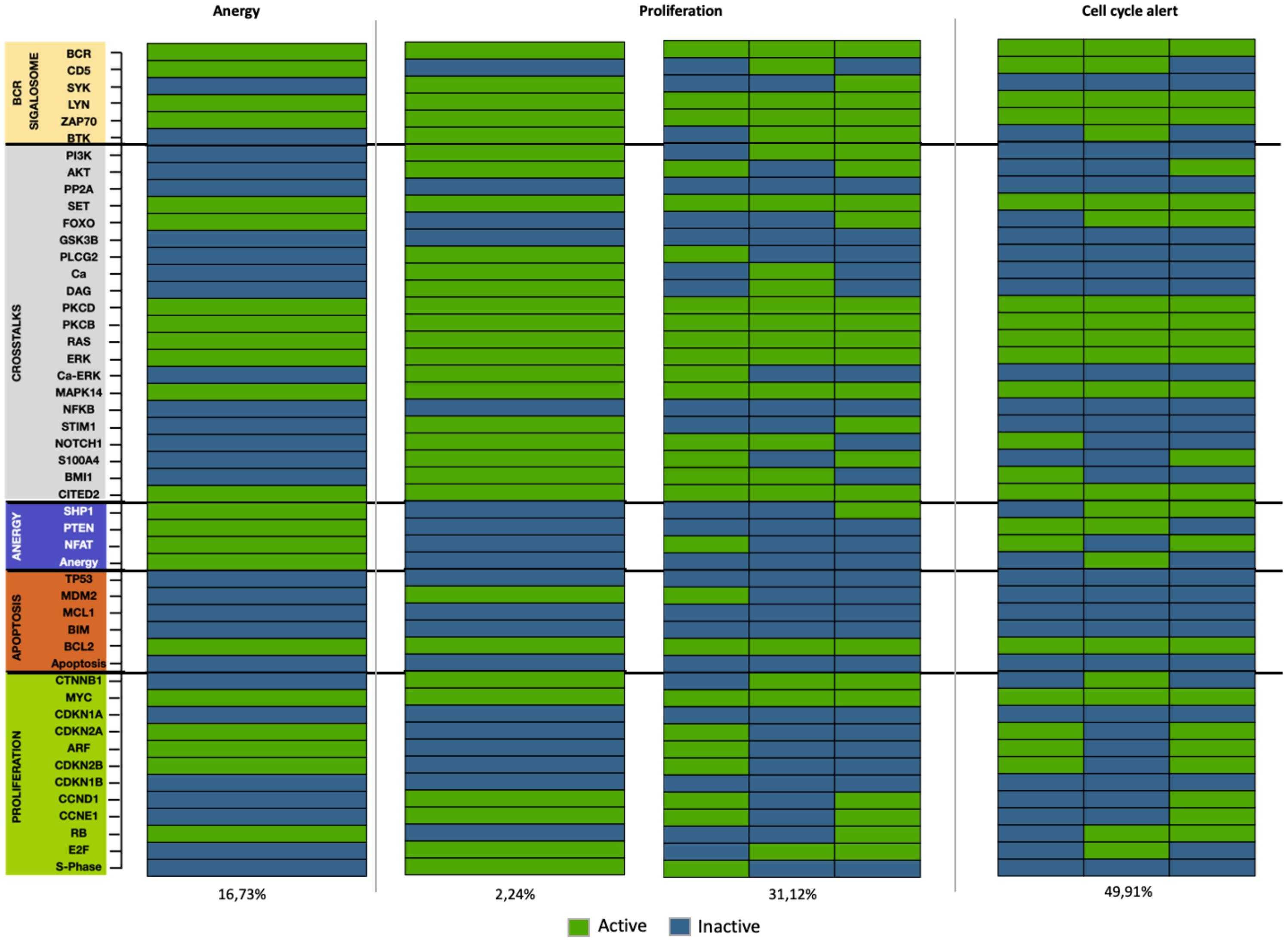

Figure S2g: Attractor pattern of the CLL model with in-silico knock-out of TP53 and knock-in of MYC and ZAP70 resembling the aberration profile of a high risk CLL. It shows one single state attractor with anergic phenotype, one single state and one cycling attractor with three states representing proliferation and one cycling attractor with three states containing one attractor with an activated cell cycle phenotype (CCND1 and CCDE1 active). The network components are listed on the left, while the state of each protein is represented by green (=active) and blue (=inactive) rectangles. Percentages below the attractor pattern shows the size of the basin of each attractor.

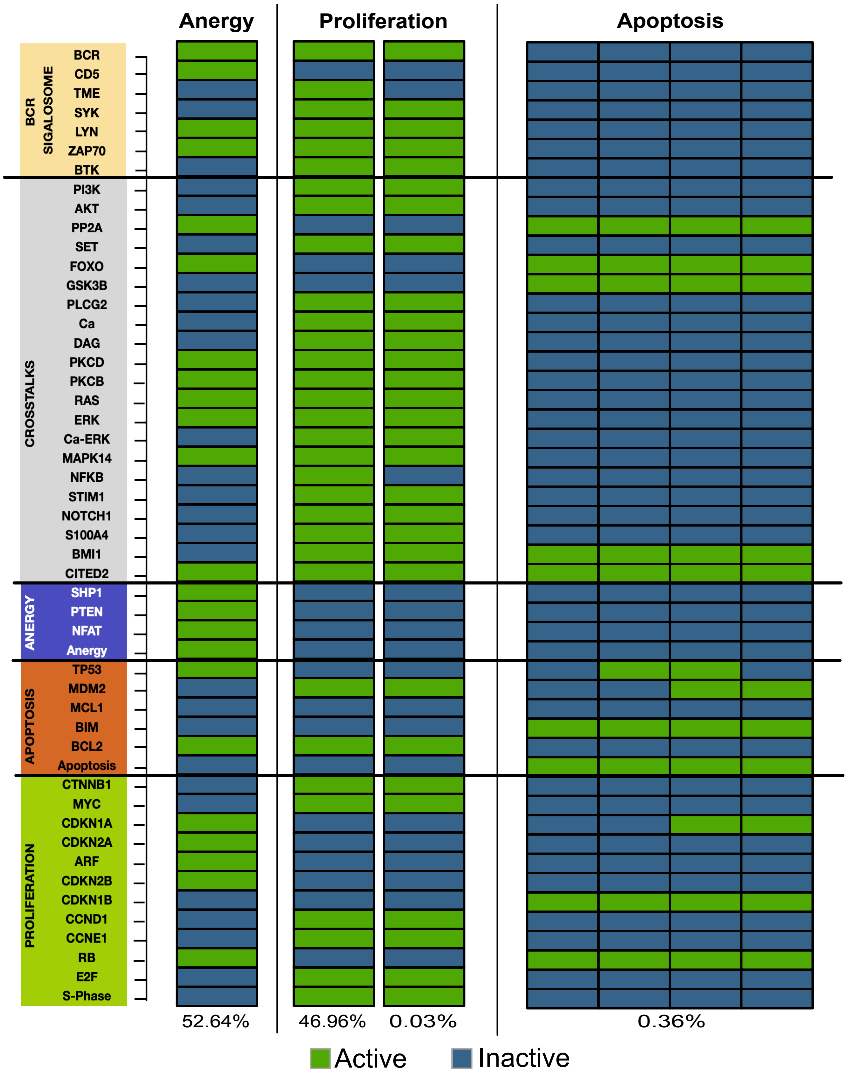

Figure S2h: Attractor pattern of the CLL integrated with TME. It shows three single state attractors representing Proliferation and Anergy, and a cycling attractor representing Apoptosis. The network components are listed on the left, while the state of each protein is represented by green (=active) and blue (=inactive) rectangles.

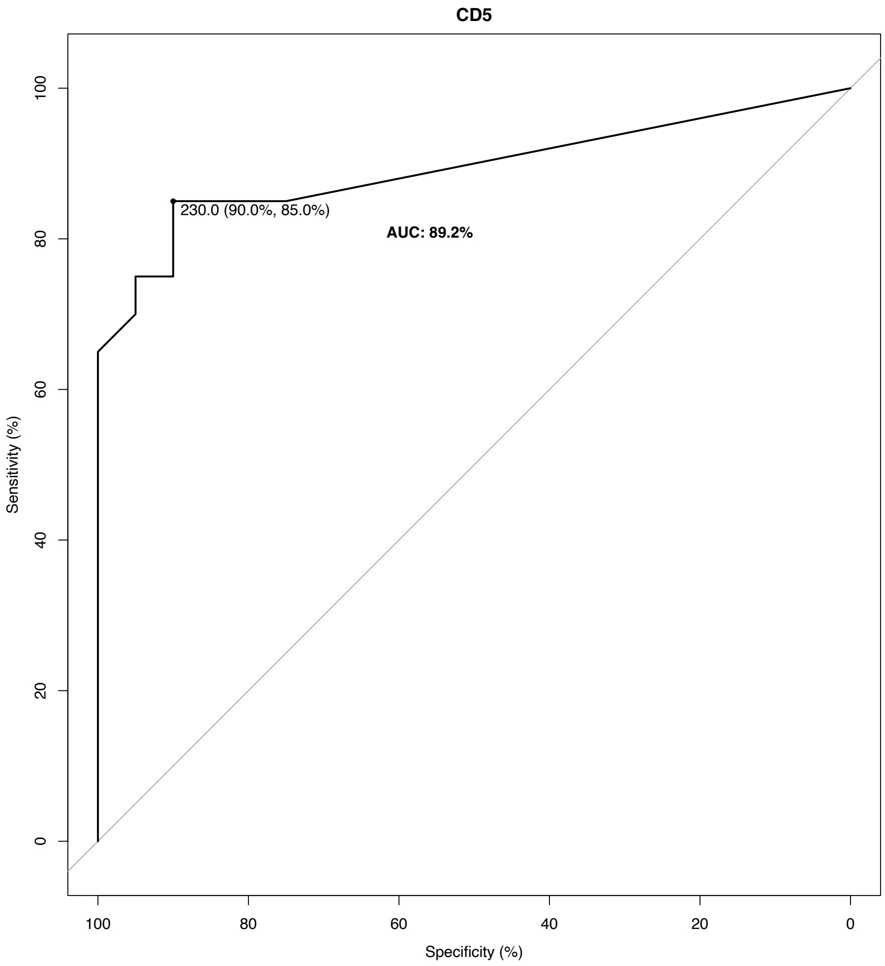

Figure S3: ROC curve for the identification of the binarization threshold of CD5. The x-axis depicts specificity in percentage, while the y-axis depicts sensitivity in percentage. The optimal threshold for specificity and sensitivity is indicated. The area under the curve (AUC) is also depicted in in the figure.

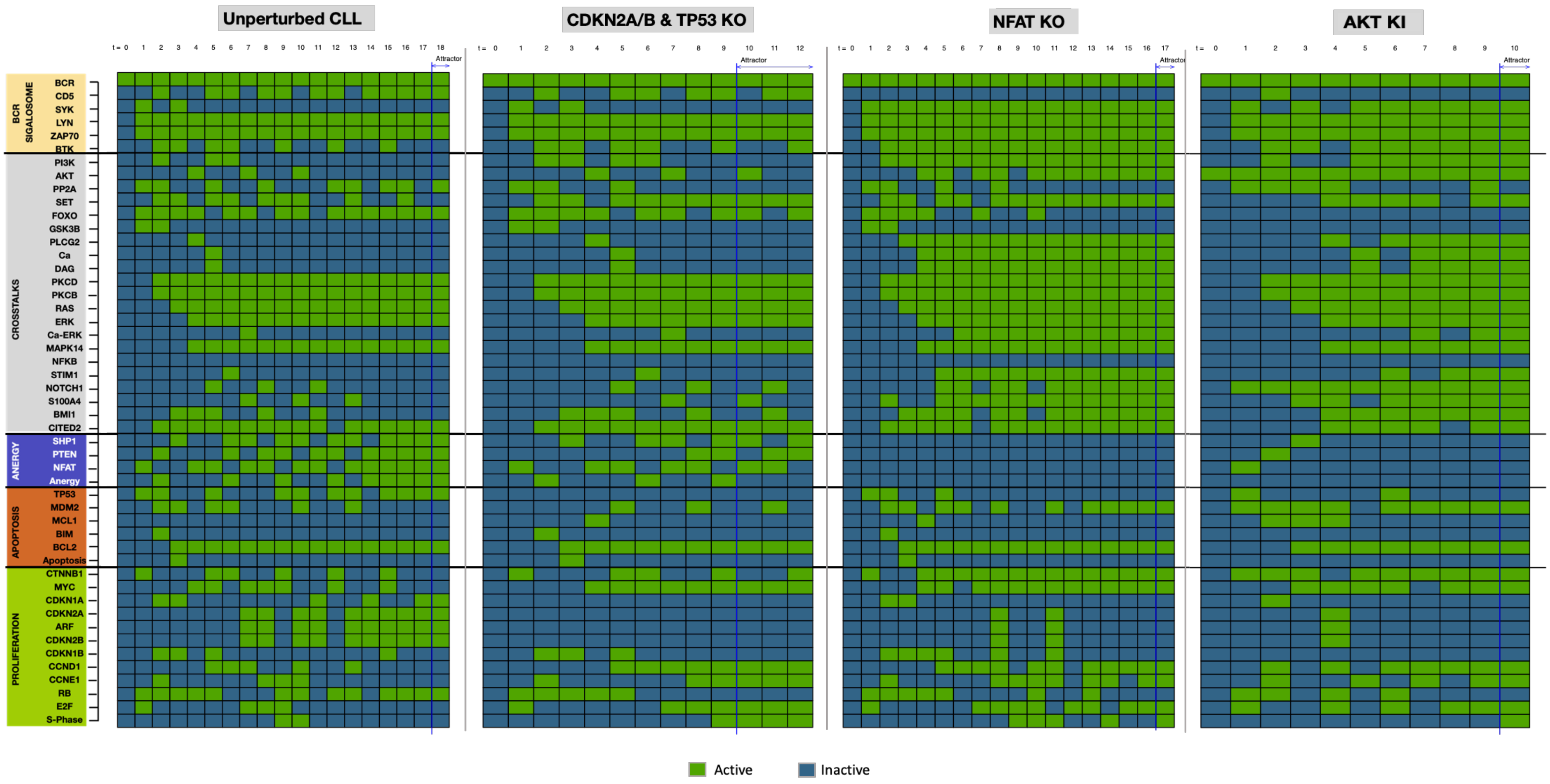

Figure S4: Simulation of a signaling cascade of the unperturbed CLL model, an in-silico knock-out of CDKN2A/B and TP53, an in-silico knock-out of NFAT and an in-silico knock-in of AKT. The signaling cascades starting from an activation of BCR are depticted and proceed in distinct time steps towards the attractors. The network components are listed on the left, while the state of each protein is represented by green (=active) and blue (=inactive) rectangles. Percentages below the attractor pattern shows the size of the basin of each attractor.

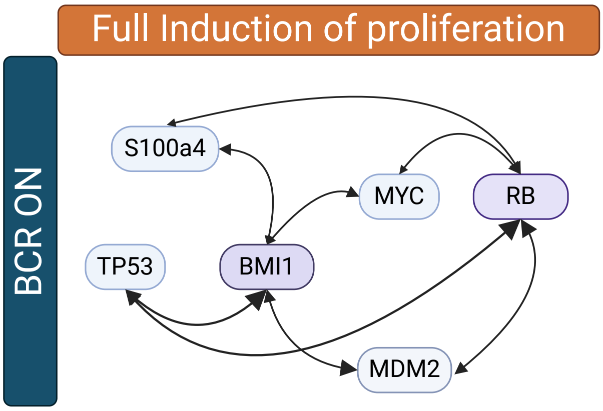

Figure S5: Tumor Driver screening results. The figure depicts the combination of drivers found by the automatic screening. Each combination returns a fully proliferative attractor landscape. Additionally, the perturbation screeening has been run under a constant BCR activation. Nodes in light purple represent drivers present in at least for combinations.

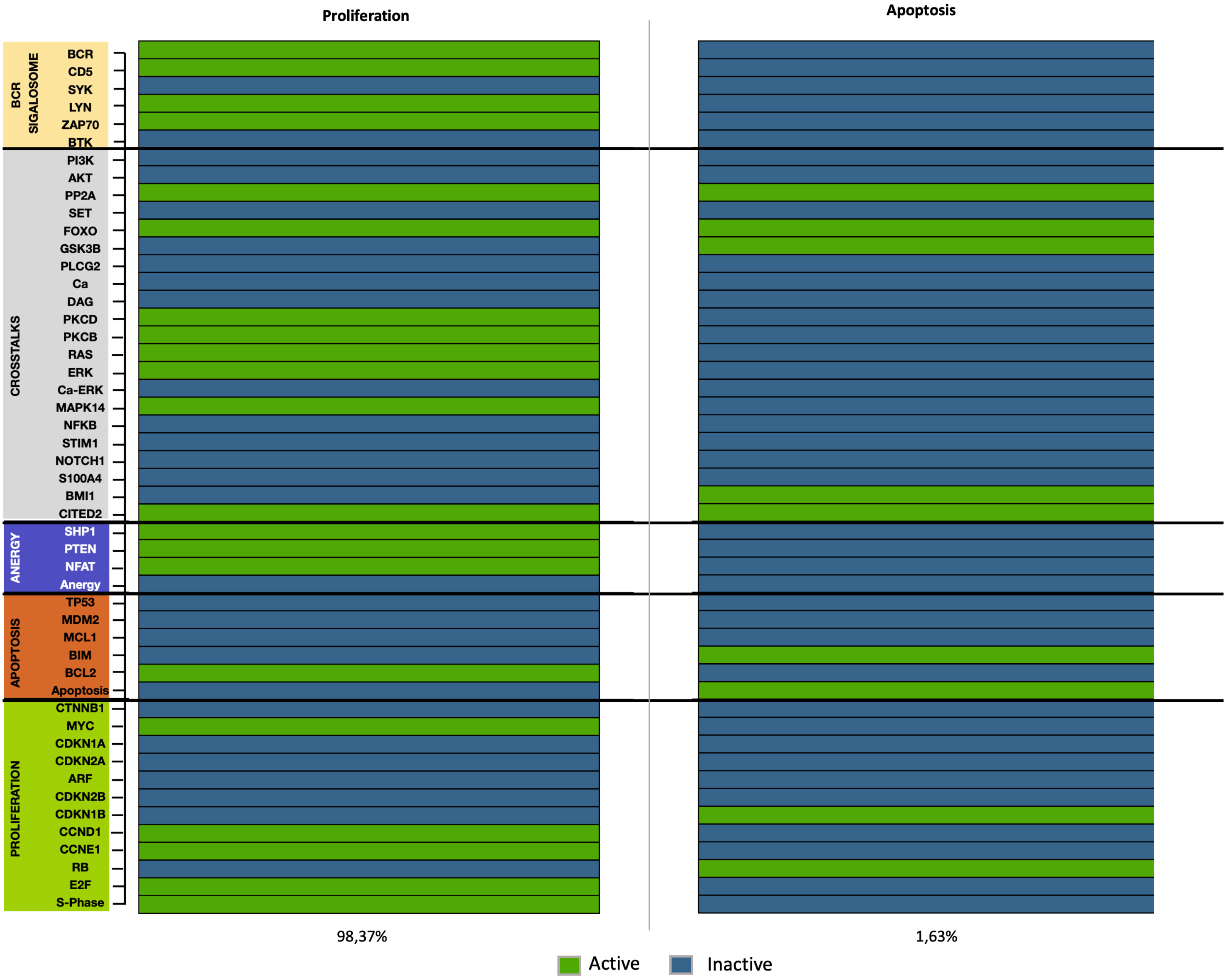

Figure S6a: Attractor pattern of an in silico knock-out of CDKN2A/B, TP53 and SET. The attractor pattern shows one single state attractor representing proliferating cells and one single state attractor with induction of apoptosis. The network components are listed on the left, while the state of each protein is represented by green (=active) and blue (=inactive) rectangles. Percentages below the attractor pattern shows the size of the basin of each attractor.

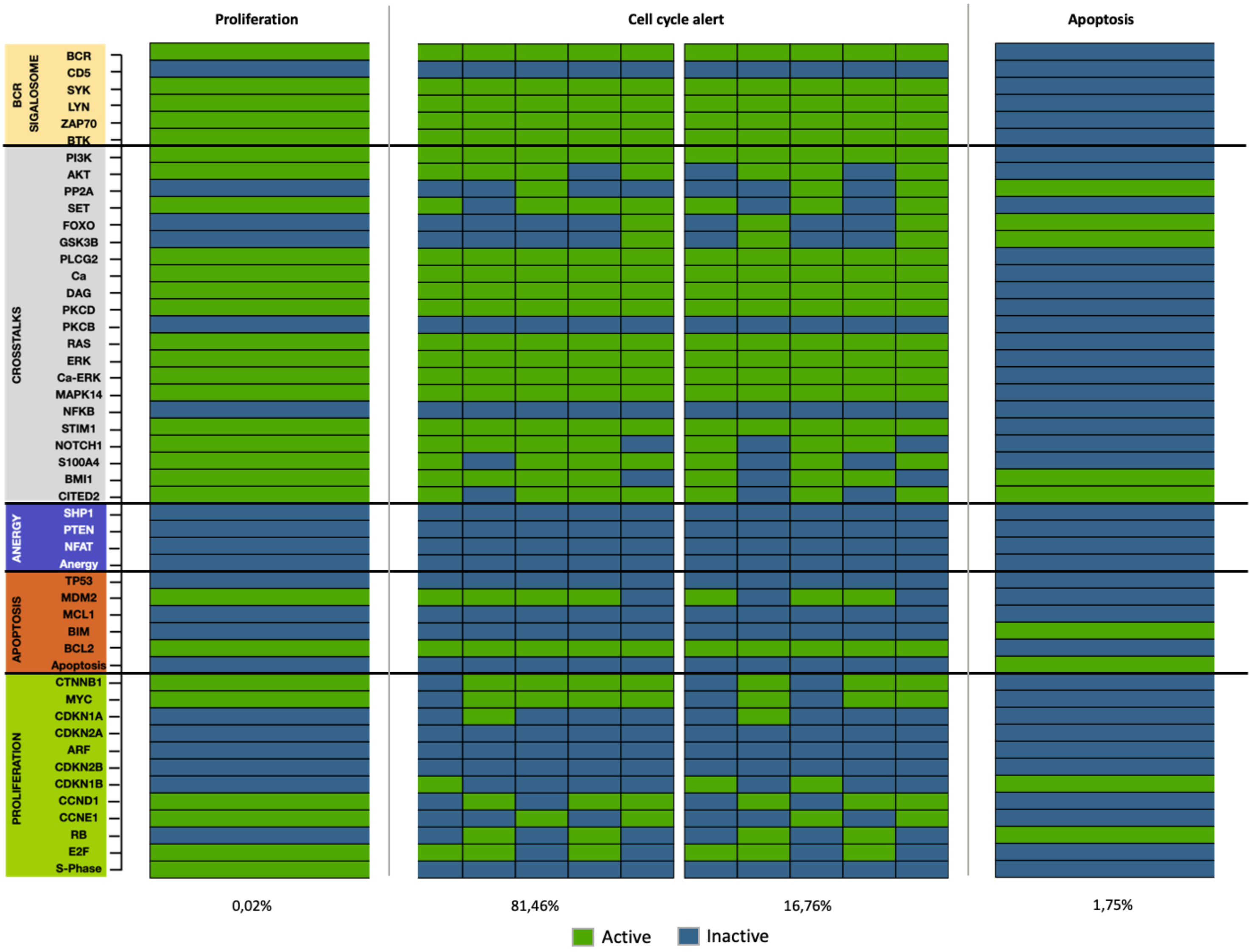

Figure S6b: Attractor pattern of an in silico knock-out of CDKN2A/B, TP53 and PKCβ. The attractor pattern shows one single state attractor representing proliferating cells, two cycling attractors with five states with a cell cycle alert phenotype and one single state attractor showing induction of apoptosis. The network components are listed on the left, while the state of each protein is represented by green (=active) and blue (=inactive) rectangles. Percentages below the attractor pattern shows the size of the basin of each attractor.

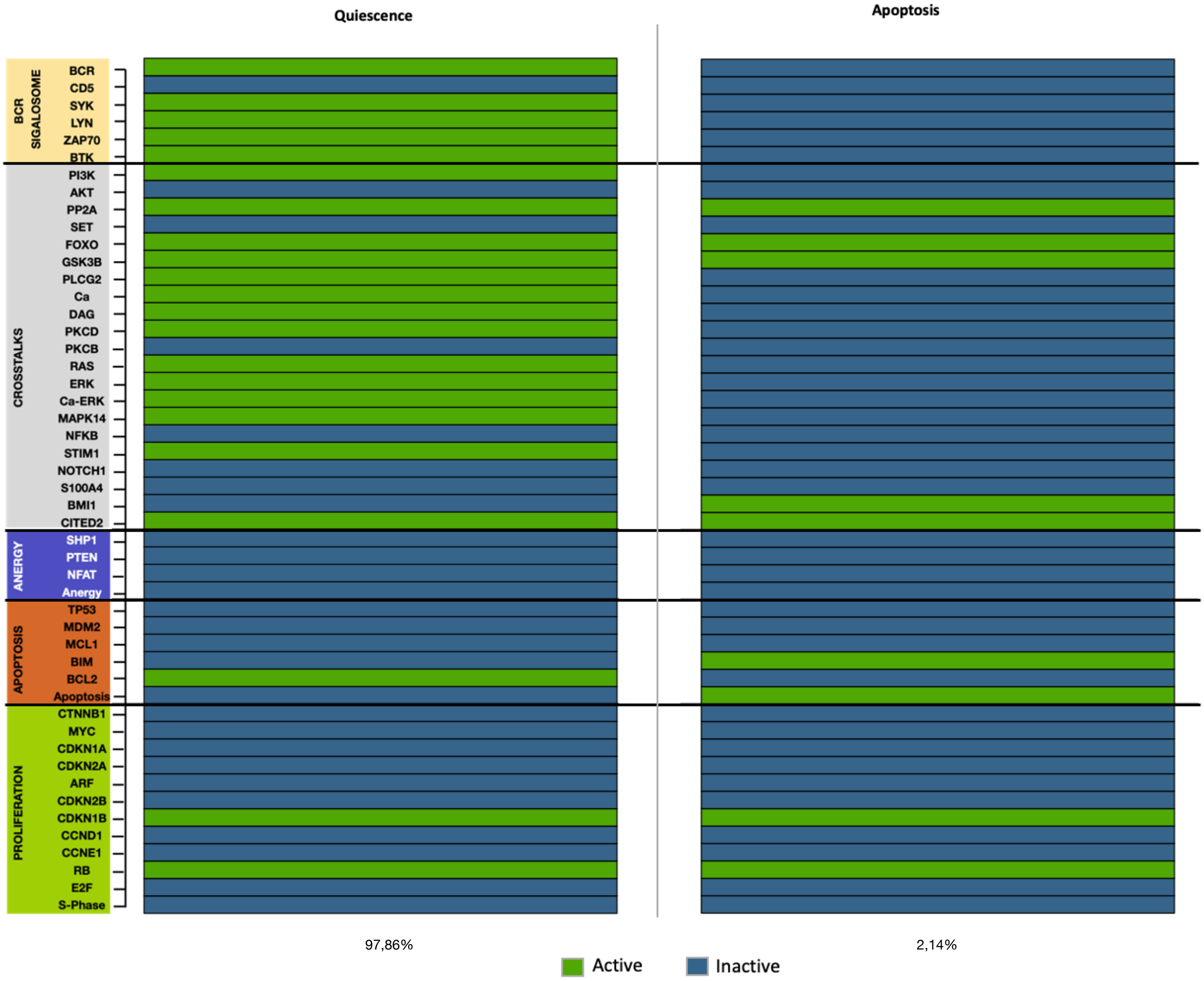

Figure S6c: Attractor pattern of an in silico knock-out of CDKN2A/B, TP53, SET and PKCβ. The attractor pattern shows one single state attractor showing quiescent cells and one with induction of apoptosis. The network components are listed on the left, while the state of each protein is represented by green (=active) and blue (=inactive) rectangles. Percentages below the attractor pattern shows the size of the basin of each attractor.

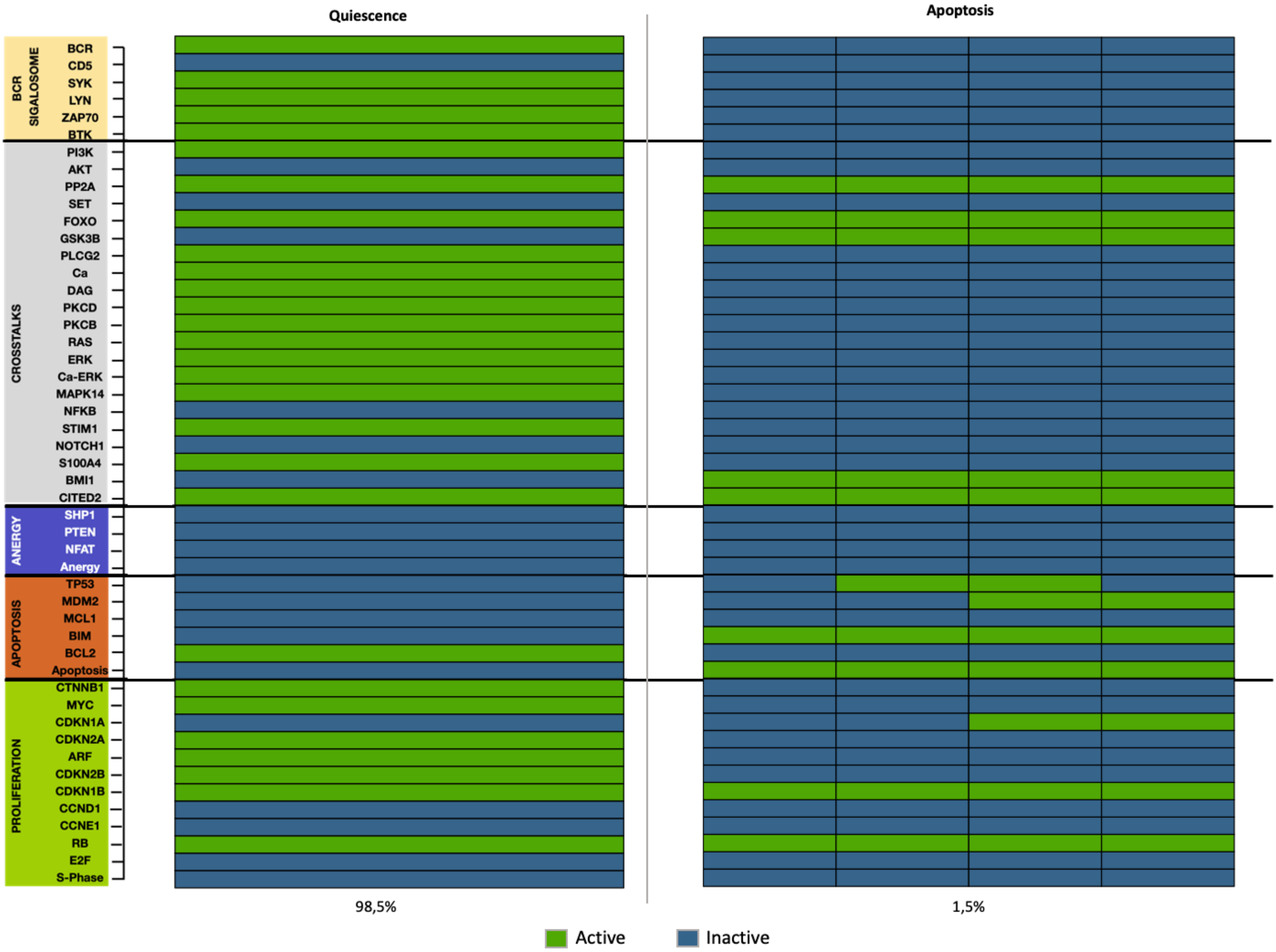

Figure S6d: Attractor pattern of an in-silico knock-out of NFAT and SET with one single state attractor representing proliferating cells and one cycling attractor showing induction of apoptosis. The network components are listed on the left, while the state of each protein is represented by green (=active) and blue (=inactive) rectangles. Percentages below the attractor pattern shows the size of the basin of each attractor.

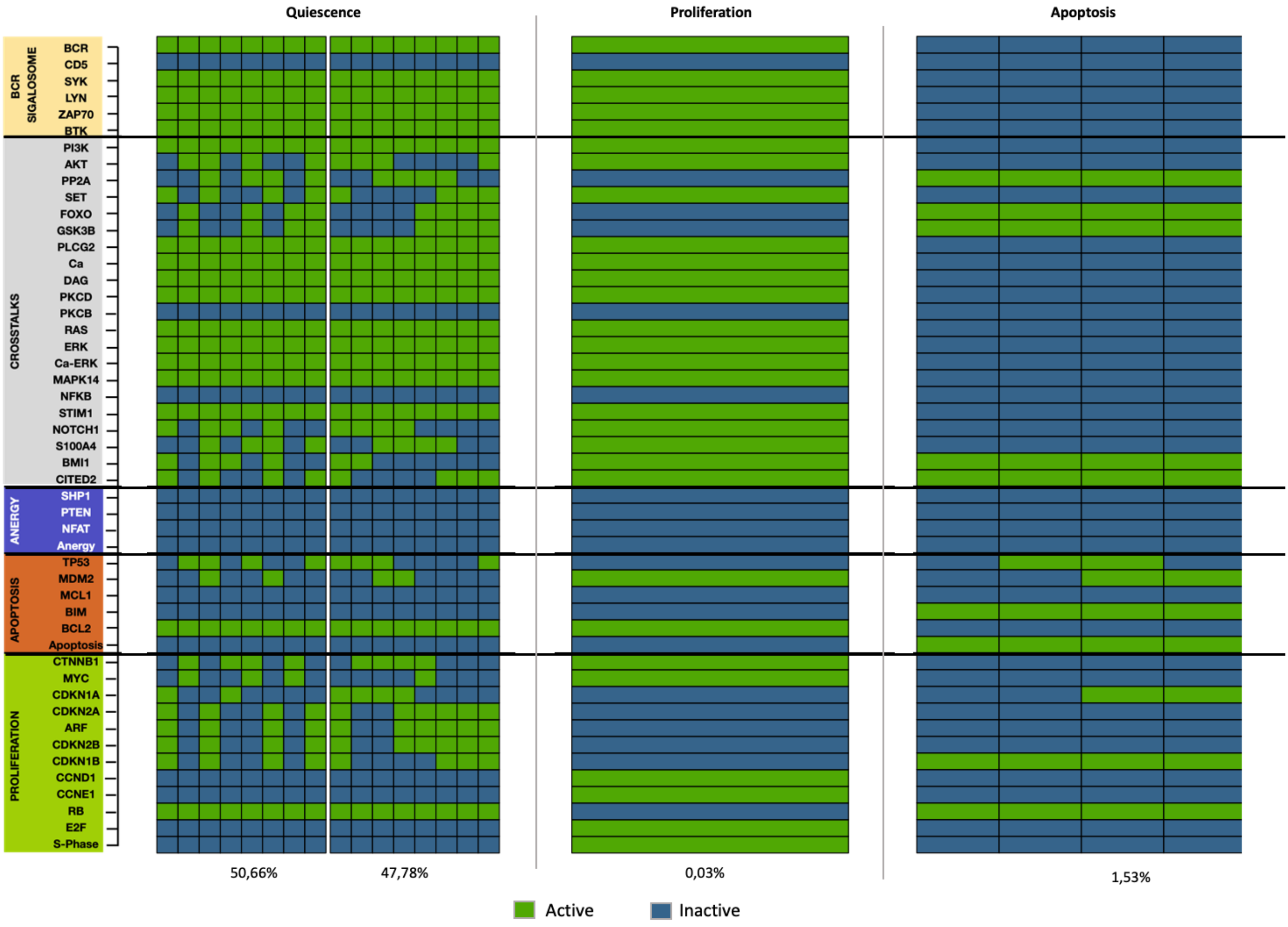

Figure S 6e: Attractor pattern of an in silico knock-out of NFAT and PKCβ. The attractor pattern shows two cycling attractors with eight states representing quiescent cells and one single state attractor showing proliferation and one cycling attractor with four states with induction of apoptosis. The network components are listed on the left, while the state of each protein is represented by green (=active) and blue (=inactive) rectangles. Percentages below the attractor pattern shows the size of the basin of each attractor.

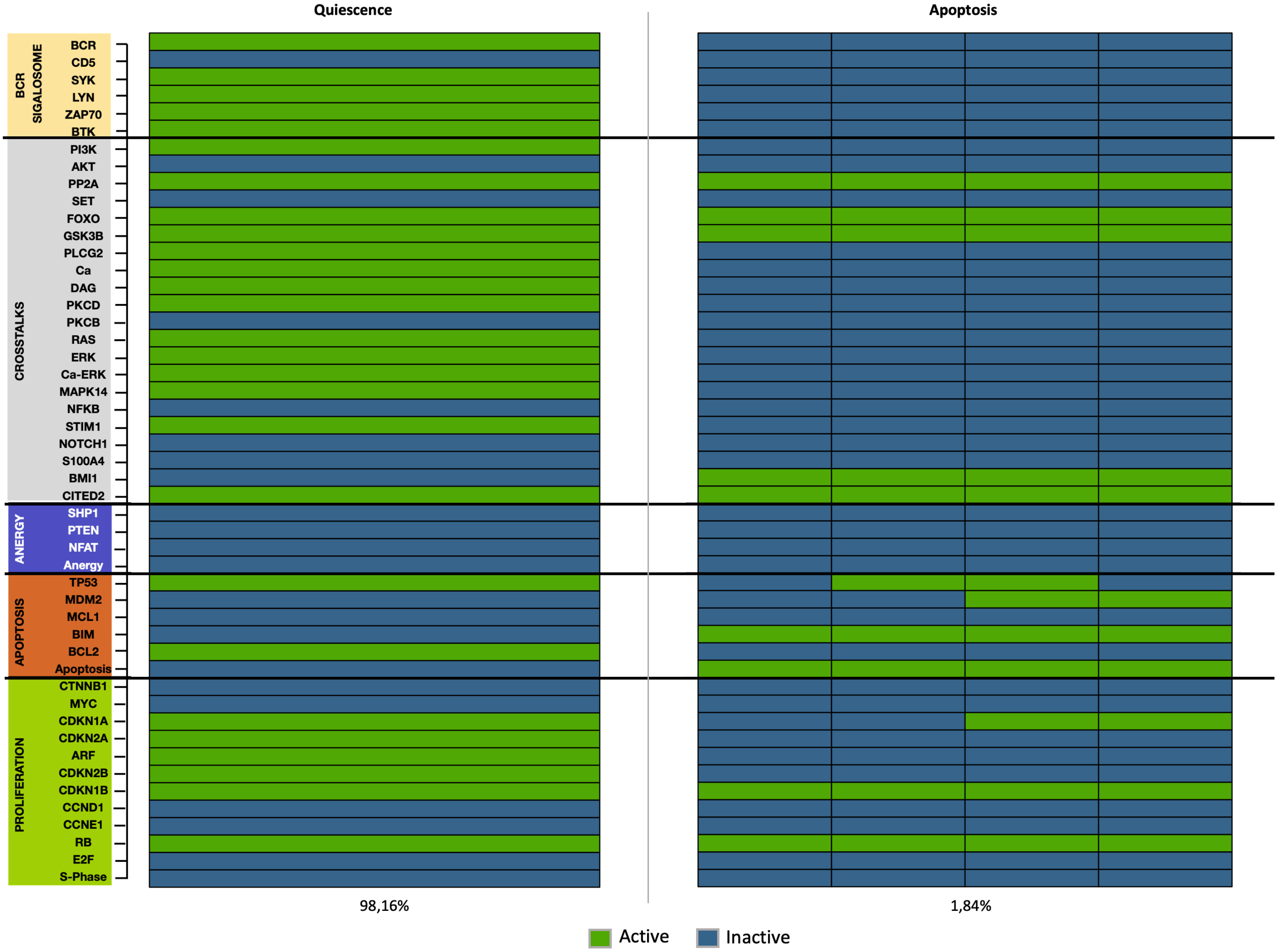

Figure S6f: Attractor pattern of an in silico knock-out of NFAT and PKCβ and SET with one single state attractor representing quiescent cells and one cycling attractor showing induction of apoptosis. The network components are listed on the left, while the state of each protein is represented by green (=active) and blue (=inactive) rectangles. Percentages below the attractor pattern shows the size of the basin of each attractor.

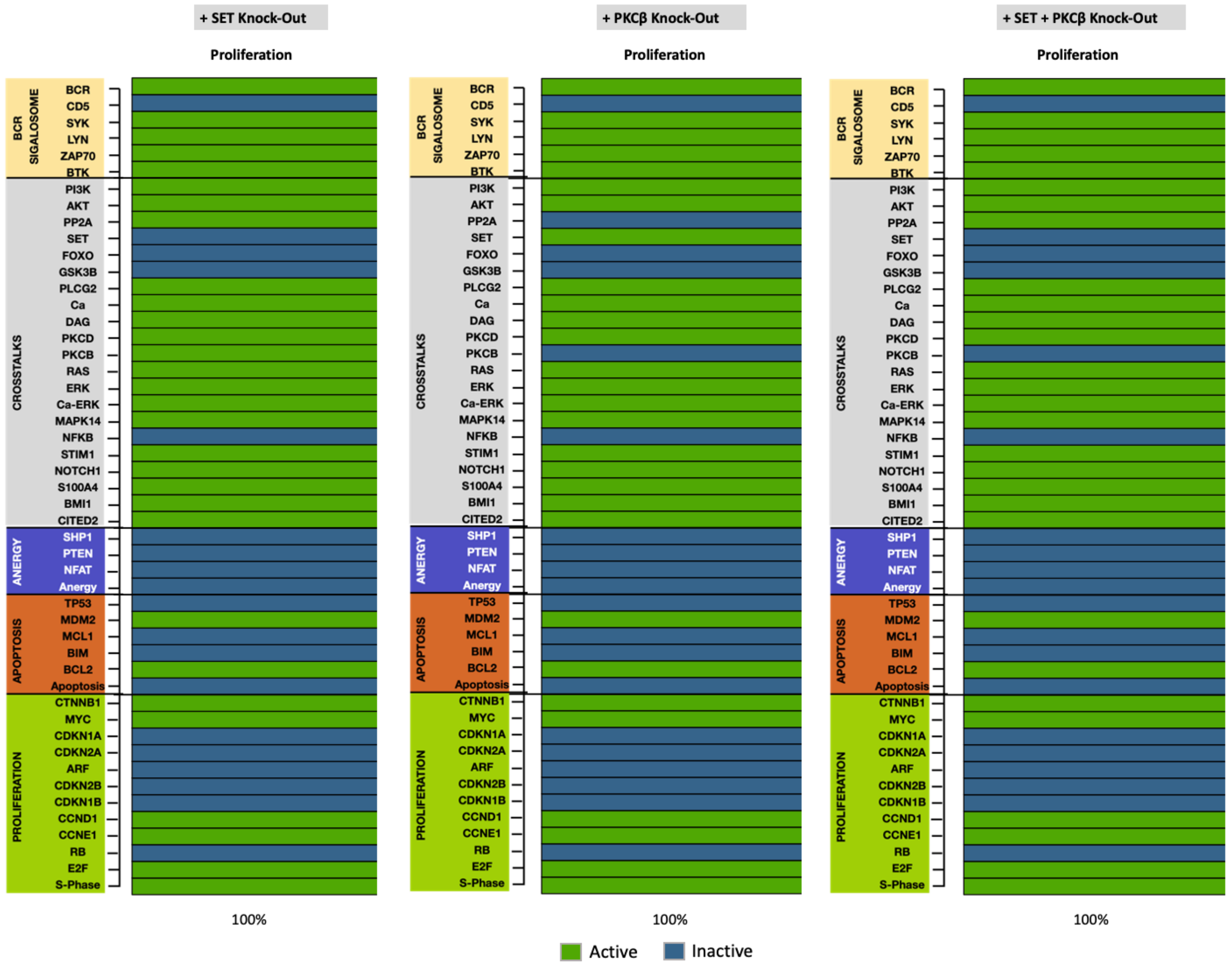

Figure S 6g: Attractor pattern of in silico knock-out of AKT in combination with knock-out of SET and/or PKCβ. All three conditions result in one single state attractor representing proliferating cells. The network components are listed on the left, while the state of each protein is represented by green (=active) and blue (=inactive) rectangles. Percentages below the attractor pattern shows the size of the basin of each attractor.

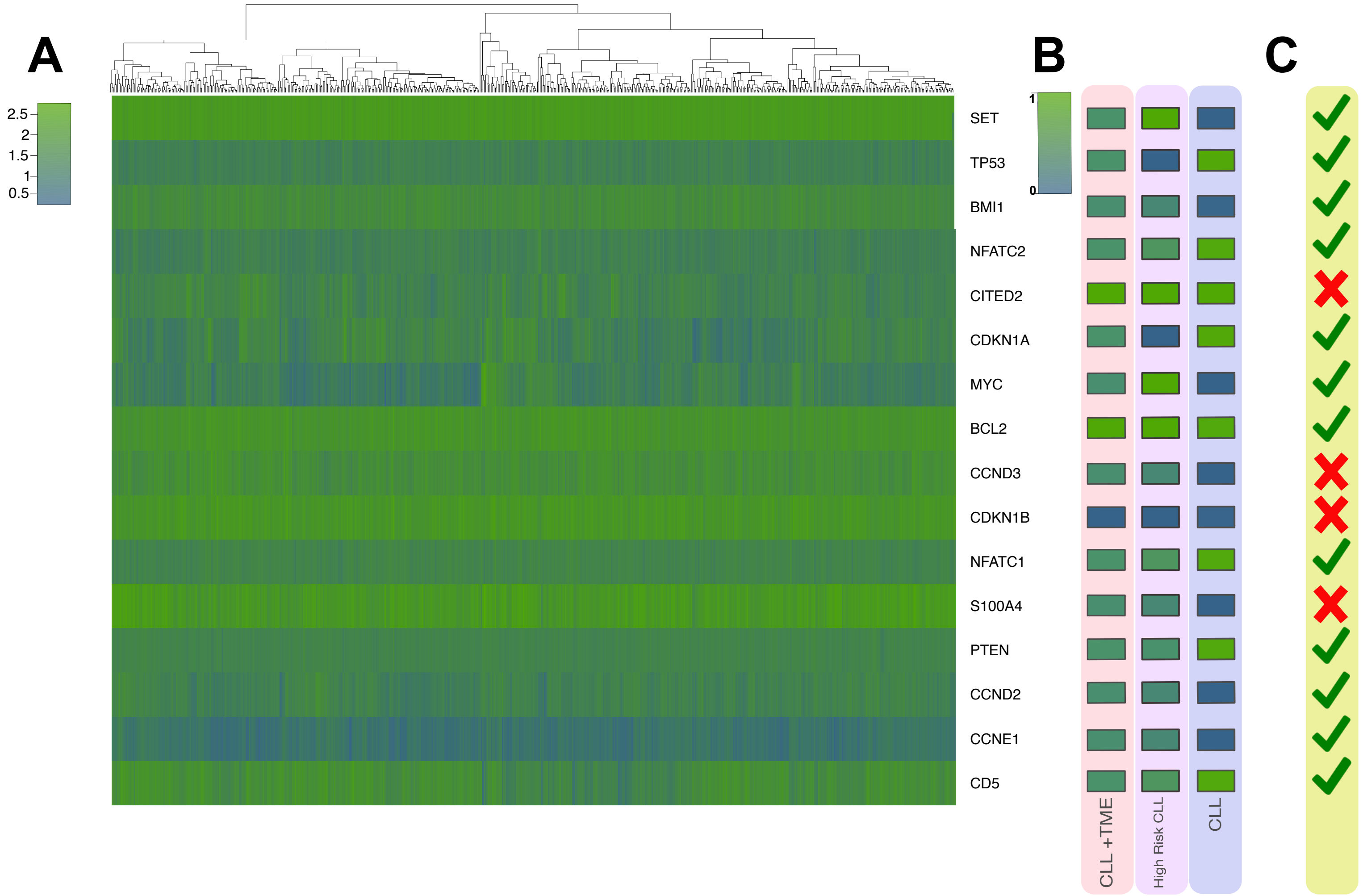

Figure S 7: Comparison of bulk RNA-seq expression values from CLLmap.org database with CLL-associated attractors from the Boolean network model. The validation is performed on the marker set of transcriptionally-regulated genes. **A)** Heatmap of tumor samples from cllmap.org (n=603). Expression values are log10 of TPM-normalized data. **B)** Averaged attractor patterns showing the activity of the three different CLL conditions simulated using the Boolean network model. **C)** Qualitative comparison of expression in dataset and average activity over the different CLL-associated attractors of the network. Mismatches were classified when no matching trends in the data could be observed. In total, we classified the attractor behavior of 12 out of 16 genes in our marker set to show the same trends in the activity pattern as can be observed in the dataset.

Supplementary Tables

Node, t+1	Boolean Function, t	Reference (Pubmed-ID)
BCR	((LYN & SYK) | ZAP70 | NOTCH1) | BCR	8349296, 12453414, 7538440, 31467429
CD5	NFAT	9647234
SYK	BCR & (BCR | LYN) & !NFAT & !SHP1	10072516, 7513017, 9857068, 12771181, 10327049
LYN	BCR & (BCR | NOTCH1)	1702903, 8349296, 1371009, 31467429
ZAP70	BCR	12393534
BTK	BCR & ((SYK | LYN) | NOTCH1 | PI3K) & !SHP1	11226282, 9188445, 31467429, 10327049, 9391111
PI3K	(LYN | SYK | ZAP70 | RAS) & !SHP1 & !PTEN	17038529, 8128248, 15536084, 20467426, 10488096, 34112805, 28480512, 9452499, 12149650, 9778245, 10339565, 1371009, 11136978, 28847905
AKT	(PKCB | PI3K) & (!LYN | (LYN & !NFAT)) & !PP2A	27494162, 20727029, 16940331, 10521450, 20079716, 15367694, 18006659
PP2A	!SET	8626647, 25931585, 21844565
SET	(GSK3B | MYC) & LYN	25931585, 21844565, 28903318, 17093053
FOXO	!AKT	10102273
PLCG2	BTK & SYK	11048639, 8691147, 8137818, 10498607, 5509800
PKCD	DAG | PI3K | SYK | LYN	12393602, 19581935, 23457006, 15105418
PKCB	Ca | DAG | PI3K | SYK | ZAP70	2479143, 19907441, 10852954, 9748166
RAS	BCR & (PKCD | PKCB | DAG)	12730099, 15899849, 15545601, 15657177, 21441934
GSK3B	!PKCB & !AKT	1324914, 17928528
NFκB	PKCB & (PKCB | NOTCH1 | PI3K | S100A4 | ERK) & !PKCD & !SHP1	16301747, 12118249, 15068587, 9712366, 22528498, 27541826, 16437144, 20669099, 15117677, 9892650, 10359702, 12870650, 25633905, 28935990, 21946908, 25633905, 20507646, 18548584 , 12835716
STIM1	Ca	21441934,19249086, 16537481, 31014395
ERK	RAS	21441934, 12835716, 15899849
Ca_ERK	STIM1 & RAS	21441934
DAG	PLCG2	11043765
Ca	PLCG2	11048639, 21441934
BMI,	(MYC | CITED2) & (!MAPK14 | (AKT & MAPK14))	21305053, 14560011, 23601184, 10541554, 21856782
CITED2	FOXO | MYC	18158893, 22814619
MAPK14	RAS	11744690, 14500381
NOTCH1	AKT | NFκB	33538798, 10329626, 22065781
S100A4	CTNNB1 & !NFAT	17101323
SHP1	LYN & CD5	21701493, 15664155, 10082557, 10540344
PTEN	(SHP1 | PTEN | TP53) & NFAT	12869565, 11545734, 21148296, 30733438
NFAT	(STIM1 | Ca | BCR) & !GSK3B & !NOTCH1	27528628, 20725108, 9072970, 20725108, 23264614, 33538798, 15866158
Anergy	NFAT &!NFκB & !AKT & !S-Phase	18292287,2897047
TP53	!MDM2 & !S100A4	9153395, 9450543, 9153396, 23752197
MDM2	(AKT | TP53) & !CDKN2A	12138177, 8440237, 8247544, 11584300, 11057904, 11923280
MCL1	(PI3K | NOTCH1 | CD5) & !GSK3B & !MAPK14	10454566, 16543145, 26041884, 16725198, 25376373
BIM	(FOXO | TP53 | Ca_ERK) & !PKCB & !BCL2 & !MCL1 & !AKT	11050388, 19907441, 22160382, 16282323, 20404322, 9430630, 20066663
BCL2	(PKCB & ZAP70) | ERK | NFκB	19907441, 12032828, 18845789, 10677502
Apoptosis	BIM & !AKT	22160382, 19713228, 10409766, 15784165
CTNNB1	!SHP1 & !GSK3B	9482734, 23778311, 8666229, 20840866
MYC	(CTNNB1 | ERK | NOTCH1 | CITED2) & !GSK3B & !TP53	22307329, 30514931, 14563837, 26629312, 19619488, 22814619, 17114293, 22461507, 11018017, 31973890, 19202062
CDKN1A	(TP53 | (RAS&E2F)) & !MYC	10702805, 35361964, 8118801, 8242752, 15616584, 11274368, 12545156
CDKN2A	RAS & !BMI1	9054499, 9923679, 10541554
ARF	RAS & !BMI1	9054499, 11057904, 10541554, 27168727, 9923679
CDKN2B	RAS & !BMI1	10733595, 10541554, 17344414, 9923679, 17055429, 20865010, 17060944, 20716961, 32428618
CDKN1B	FOXO & !SHP1	10827191, 18234419, 19838216
CCND1	(NOTCH1 | NFκB | CTNNB1 | MYC) & !CDKN2B & !CDKN2A & !CDKN1A & !GSK3B	10082535, 11312120, 10409765, 10318916, 15067010, 15752976, 22960768, 18023328, 9832503, 7626805, 19001083, 22173954, 20562911, 19001083, 28314854, 20887720
CCNE1	E2F & ((!CDKN1A & !CDKN1B)| CCND1)	8288131, 10385618, 16330818, 11937641, 8843201
Rb	!CCND1	8939849, 11250068, 9529601
E2F	!CDKN1A & !Rb	10498892
S-Phase	CCNE1 & E2F	27084253, 22123859

Table S1: Boolean functions of the CLL model. Depicted are the Boolean functions of the analyzed model. Interactions are described by logical connectives AND (&), OR (|), and NOT (!).

Input node	Related Pathway	Reference
BAFF-R	NFκB	27756161, 16973958
TACI, BCMA	NFκB	27756161, 16973958
CD40, CD154	NFκB	11967542, 23760533, 19426221, 15175625
TLR	via TRAF6: NFκB	27756161, 19685493, 17768129, 30498085
Interleukine /Chemokine Receptors	AKT, JAK/STAT, NFκB	27756161, 11023495

Table S2: Integration of literature for TME.

Node	New rule	Reference
NFKB	TME | (PKCbeta & (PKCbeta | NOTCH1 | PI3K | S100a4 | ERK) & !SHP1 & !PKCdelta)	27756161,16973958, 16973958, 11967542, 23760533, 19426221, 15175625, 19685493, 17768129, 30498085
AKT	TME | ((PKCbeta | PI3K) & (!LYN | (LYN & !NFAT)) & !PP2A)	11967542, 23760533, 19426221, 15175625, 27756161, 11023495
BCR	TME | (((LYN & SYK) | ZAP70 | NOTCH1) | BCR)	Model Assumption

Table S3: Extension of the CLL model integrating the TME.

Entity	Pat ID	CD5	BMI1	TP53	Ki67
CLL	Pt20	300	NA	NA	NA
CLL	Pt21	300	NA	NA	NA
CLL	Pt22	300	NA	NA	NA
CLL	Pt23	260	NA	NA	NA
CLL	Pt24	300	NA	NA	NA
CLL	Pt25	300	NA	NA	NA
CLL	Pt26	280	NA	NA	NA
CLL	Pt27	300	NA	NA	NA
CLL	Pt28	260	NA	NA	NA
CLL	Pt29	60	180	1	3
CLL	Pt30	300	NA	NA	NA
CLL	Pt31	100	200	1	5
CLL	Pt32	300	NA	NA	NA
CLL	Pt33	300	NA	NA	NA
CLL	Pt34	300	NA	NA	NA
CLL	Pt35	300	NA	NA	NA
CLL	Pt36	300	NA	NA	NA
CLL	Pt37	300	NA	NA	NA
CLL	Pt38	300	140	1	15
CLL	Pt39	NA	20	0	2
CLL	Pt40	NA	130	1	17
CLL	Pt41	NA	90	1	20
CLL	Pt42	NA	100	1	2
CLL	Pt43	NA	145	1	5
CLL	Pt44	NA	140	1	7
CLL	Pt48	NA	60	0	3
CLL	Pt49	NA	30	0	10
CLL	Pt8	300	30	1	3
CLL	Pt8	300	80	0	60
RS	Pt1	60	150	0	60
RS	Pt10	20	200	1	70
RS	Pt11	0	130	1	45
RS	Pt12	70	NA	NA	NA
RS	Pt13	0	NA	NA	NA
RS	Pt14	0	NA	NA	NA
RS	Pt15	0	NA	NA	NA
RS	Pt16	0	NA	NA	NA
RS	Pt17	20	NA	NA	NA
RS	Pt18	200	NA	NA	NA
RS	Pt19	300	NA	NA	NA
RS	Pt2	300	50	1	70
RS	Pt3	300	250	1	80
RS	Pt4	0	180	1	50
RS	Pt45	NA	90	1	70
RS	Pt46	NA	110	1	80
RS	Pt47	NA	160	0	100
RS	Pt5	0	140	0	70
RS	Pt6	140	150	0	65
RS	Pt7	0	180	0	90
RS	Pt8	0	30	1	3
RS	Pt8	0	80	0	60
RS	Pt9	0	180	0	40
RS	Pt9	0	180	0	40

Table S4: List of specific patient information from the established cohort of CLL and RS cases.

Target	Nr Clinical Trial Drugs	Phase	Nr of patented Drugs/ Preclinical	Nr Discontinued Drugs	Type	Oncology	Leukemia	CLL	RS	Not Oncology	Disease of application
BCR	2	Approved	None	None	Small Molecule	YES	YES	YES			Chronic Lymphocytic Leukemia
CD5	1	Phase I	None	None	Car-T Cell therapy	YES	YES	YES			Chronic Lymphocytic Leukemia; CD5+ Relapsed/refractory Malignancies
SYK	18	Approved/Phase I/II/III	None	2	Small Molecule	YES	YES	YES	YES	YES	Immune Thrombocytopenic Purpura (Approved), Thormbocytopenia, B-cell Lymphoma, Reumatoid arthritis, Cutaneous Lupus Erythematosus, Diffuse large B-cell Lymphoma, Non-Hodgkin Lymphoma, Acute Myeloid Leukaemia, Asthma, Chronic Lymphocytic leukaemia, Solid Tumor, Chronic obstructive Pulmonary Disease, Eczema
LYN	2	Phase I/II	None	None	Small Molecule	YES	YES	YES		YES	Bone Disease, Solid Tumor, Chronic Lymphocytic Leukaemia
ZAP70	0	None	1	None	Small molecule						Not available
BTK	1	Approved	None	None	Small Molecule	YES	YES	YES	YES		Leukemia, Chronić Lymphocitic Leukemia, Richter Syndrome
PI3K	11	Approved/Phase I/II/III	None	None	Small Molecule	YES	YES	YES	YES		Follicular Lymphoma, Breast Cancer, Solid Tumor, Colorectal Cancer, Chornic Lymphocytic Leukaemia, Richter Syndrome
AKT	13	Approved	None	1	Small Molecule/Combinatory Drug	YES	YES	YES		YES	Breast Cancer (Approved), Non-Hodgkin Lymphoma, Proteus syndrome, Lymphoma, Myocardial Perfusion Injury, Leukemia, Parkinson Disease, Solid Tumor, Artheriosclerosis
PP2A	2	Phase II	None	None	Small Molecule	YES				YES	Astrocytoma, Molluscus contagiosum infection
SET	None	None	1 (PMIDs: 21844565, 34244560, 25763353)	None	Small Molecule	YES	YES				B-cell Malignancies, Breast Cancer, Leukaemia
GSK3B	6	Phase II/III	3	2	Small Molecule/Receptor Modulator	YES	YES			YES	Myotonic Dystrophy, Myelofibrosis, Fragile X syndrome, Acute Myeloid Leukaemia, Osteosarcoma, Parkinson Disease, Ovarian Cancer, Graft Rejection, Malignant Glioma, Alzheimer Disease
PKCB	5	Phase I/II/III	None	1	Small Molecule	YES	YES	YES		YES	Non-Hodkin Lymphoma, Lymphoma, Solid Tumors, Renal Transplantation, Chronic Lymphocytic Leukemia, Rhinitis
RAS	4	Phase II	None	1	Small Molecule/Vaccine/Reovirus	YES					Colorectal Cancer, Head and Neck Cancer, Lung Cancer, Solid Tumors
ERK	7	Phase I/II	1	1	Small Molecule/ Drug Eluiting Stent	YES	YES	YES		YES	Melanoma, Solid Tumor/Cancer, Artery Stenosis, T-cell Leukemia, Chronic lymphocytic Leukemia
MAPK14	5	Approved (Phase IV)/Phase II/III	None	7	Small Molecule	YES				YES	Dry Eye Syndrome (Approved), Acute Coronary Syndrome, Acute Lung Injury, Rheumatoid arthritis, Chronic Obstructive Polmonary Disease, Psoriasis Vulgaris, Solid tumors
NFKB	14	Approved/Phase I/II/III	17	5	Small Molecule/Decoy (Modulator Drug)	YES	YES			YES	Rheumatoid arthritis (Approved), Duchenne Dystrophy, Lupus, Multiple Sclerosis, Autoimmune Diabetes, Fungal infection, Acute Liver Failure, Atopic Dermatitis, Inflammatory Bowel Disease, Solid Tumors, Acute Myeloid Leukaemia, Skin Infection, Multiple Myeloma, Gynecological Disease, Asthma, Cyctic Fibrosis
STIM1	None	None	2 (PMIDs: 27726010, 29337250)	None	Small Molecule					YES	Heart Hyperthorpy and Injury
NOTCH1	2	Phase I/II	None	None	Small Molecule, Antibody	YES	YES				T-cell Acute Lymphoblastic Leukaemia, Solid Tumors
S100A4	None	None	1	None	Small Molecule	YES					Breast Cancer
BMI1	1	Phase II/III	None	None	Small Molecule	YES					Leiomyosarcoma
TP53	17	Phase I/II/III	None	None	Small Molecule/siRNA/Gene Therapy/Vaccine/Adnovirus/Antibody	YES	YES			YES	Oral Cancer, Renal Transplantation, Polycystic Ovary Syndrome, Solid Tumor, Acute Myeloid Leukemia, Prolymphocitic Leukemia, Ovarian Cancer, Head and Neck Cancer, Pancreatic Cancer, Colorectal Cancer, Cervical Cancer,
MDM2	10	Phase I/II/III	None	None	Small Molecule	YES	YES	YES			Solid tumors, Heamatological Malignancy, Merkel Cell Carcinoma, Proymphocitic Leukaemia, Acute myeloid leukaemia, Prostate Cancer
MCL1	7	Approved/Phase I/II	None	2	Small Molecule	YES	YES	YES		YES	Chronic Mylogenous Leukemia (Approved); Solid tumor, Multiple Myeloma, Refractory haematological malignancy, Acute Myeloid Leukaemia, Melanoma, Trematode Infection, Asthma
BCL2	21	Approved/Phase I/II/III	None	None	Small Molecule	YES	YES	YES	YES	YES	Chronic Lymphocytic Leukemia (Approved), Amyotrophic Lateral Sclerosis (Approved), Solid Tumor (Approved), Myelofibrosis, Multiple Myeloma, Breast Cancer, Polycystic Ovary Syndrome, Prostate Cancer, Small Cell Lung Cancer, Follicular Lymphoma, Acute Myeloid Leukaemia, Psoriasis, Multiple Myeloma
CTNNB1	4	Approved/ Phase I/II	None	None	Recombinant Human Endostatin/ siRNA/Small Molecule	YES					Solid Tumors (Approved), Scleroderma, Familiar Adenomatous Polyposis, Desmoid Tumor
MYC	2	Phase II	None	None	Antisense Drug					YES	Coronary Artery Disease
CCND1	3	Phase I/II/III	None	1	Small Molecule					YES	Myelofibrosis, Solid Tumor, constitutional Neutropenia, platelet aggregation disorder
CCNE1	None	None	None	1	Small Molecule	YES					Retinoblastoma
E2F	1	Phse I	None	1	Small Molecule/antisense Drug	YES				YES	Solid Tumors, Platelet Aggregation disorder

Table S5: Detailed screening of targetable nodes in the CLL model. For each target node in the CLL model the number of drugs in either clinical, preclinical, or withdrawn state are reported according to the TTD screening. Additionally, the type of drugs and the application areas is reported. As areas oncology (further specified as leukemia, CLL, RS) and not oncology applications. were considered The specific designated diseases for each node target are reported. The results are integrated with the screening of clinicalTrials.gov for CLL and RS specific clinical trials. Results for each target node are comulative for the reported set of existing drugs in the TTD or further expanded by literature screening for drugs. Nodes in the CLL model without a hit neither in the TTD nor in the literature screening are not included in the table.
